# Haptic feedback in violin education as a case study of robotic exoskeleton-mediated motor learning

**DOI:** 10.1038/s41598-026-39226-8

**Published:** 2026-03-04

**Authors:** Adriaan Campo, Emanuele Peperoni, Stefano Laszlo Capitani, Roberto Conti, Simona Crea, Nicola Di Stefano, Francesco Di Tommaso, Francesco Giovacchini, Canan Gener, Lorenzo Grazi, Luca Morelli, Alessia Noccaro, Andrea Parri, Nevio Luigi Tagliamonte, Emilio Trigili, Nicola Vitiello, Shalan Alhamwy, Domenico Formica, Marc Leman

**Affiliations:** 1 Faculty of Arts and Philosophy, Institute of Psychoacoustics and Electronic Music (IPEM), Ghent University, Ghent, Belgium; 2 The BioRobotics Institute, Department of Excellence in Robotics and AI, Scuola Superiore Sant’Anna, Pisa, Italy; 3 IUVO S.r.l, Pontedera, Pisa, Italy; 4 Institute of Cognitive Sciences and Technologies (ISTC), National Research Council (CNR), Rome, Italy; 5 Università Campus Bio-Medico di Roma, Rome, Italy; 6 Neurorobotics Lab, Newcastle University, Newcastle upon Tyne, UK; 7 De Centrale, Ghent, Belgium

**Keywords:** Biomedical engineering, Neuroscience

## Abstract

This study explores the use of an upper-limb wearable exoskeleton for teaching violin technique to novices through haptic-assisted training. The educational potential of this technology was evaluated using a framework that integrated quantitative kinematic metrics and qualitative assessments, comparing a group receiving haptic feedback (*N* = 12) with a control group that did not (*N* = 12) in a mixed between-within-subjects design. Results from a double-blind expert panel indicated that participants trained with the exoskeleton outperformed the control group during recall measurements. In addition, spatial and spatiotemporal - but not temporal - kinematic metrics related to bowing technique improved significantly during training, with gains persisting during recall, and these outcomes were supported by self-reported user assessments. However, subtle features of the technology also became apparent, including interference with natural shoulder movements, exposing areas for refinement. In conclusion, this study suggests that haptic-assisted training with an exoskeleton can enhance specific motor skills in violin playing, highlighting promising directions for future research in educational technology and providing a quantitative framework for assessing motor skill development in haptic-assisted learning, which can further support the development and validation of educational technologies. However, given the absence of long-term retention measurements and the limited generalizability associated with the small sample size, the findings of this pilot study should be interpreted with caution.

**Supplementary Information:**

The online version contains supplementary material available at 10.1038/s41598-026-39226-8.

## Introduction

In recent years, technological advancements have enabled the use of sensory feedback tools, including visual, auditory, and haptic cues, to enhance motor learning and support neurorehabilitation^1–4^. Studies have shown that haptic feedback in human-human interaction can improve task performance and motor learning^5^. Vibrotactile cues, e.g., delivered through wearable devices can enhance performance in simple sports and dance tasks, although designing effective stimuli for complex movements remains challenging^6^. Conversely, joint-torque feedback provides intuitive, movement-related information with neurophysiological benefits, yet evidence of its superiority over visual cues for joint coordination is still limited^7–9^. Robotic systems can further mediate such feedback between distant partners, enabling human-robot-human (HRH) interaction^10^.

Despite this growing body of research on robot-assisted learning, few studies have examined the potential of such technology in music education. The present study investigates the learning and performance outcomes of using an upper-limb wearable robot to teach violin technique to novices. To our knowledge, it is the first to explore haptic-assisted music training through both qualitative and quantitative measures in comparison with a control group.

### Haptic-Assisted training

The effectiveness of haptic-assisted training has been widely investigated in the fields of motor learning and neurorehabilitation. However, findings across studies remain inconclusive, likely due to variations in motor tasks, experimental protocols, haptic modalities, and participant skill levels^11^. In general, however, haptic methods tend to outperform non-guided training^12^ and are often at least as effective as audiovisual or auditory feedback in teaching the spatial^13^, temporal^14–16^, and spatiotemporal^16^ aspects of motor tasks.

In addition, haptic assistance has been shown to significantly reduce spatial errors and error variability during transfer and retention tests compared to unguided training in tasks such as trajectory following and line tracking^17,18^. In the temporal domain, haptic methods have proven effective in improving learning outcomes relative to unguided training, notably reducing timing errors over both short and long durations in activities such as driving, steering, tennis, and rowing^19–21^. Similarly, in the spatiotemporal domain, performance-enhancing haptic feedback has improved execution in tasks such as golf putting, rowing, and circle drawing^22,23^.

Interestingly, the benefits of haptic-assisted training are not consistently observed in simpler tasks^20,24,25^ or among highly skilled participants who are already proficient in the task^26^. This suggests that while haptic guidance can facilitate learning by reducing task difficulty to an optimal level, it hinder skill acquisition when the task already lies within the learner’s capability range. Therefore, aligning task complexity with participants’ skill levels is critical for maximizing the effectiveness of haptic training^27^.

Haptic interaction is thus particularly relevant in complex domains such as music education, where physical interaction among teacher, student, and instrument is fundamental^28^. For instance, the HAGUS apparatus^16^ demonstrated that audio-haptic demonstration in a percussion task significantly improved spatiotemporal performance compared to haptic or auditory feedback alone. Similarly, the MoveMe system^29^ provided prerecorded haptic trajectories to support violin training. Both approaches offer proof of concept for integrating haptic feedback into music education.

### Metrics for violin playing performance

To validate the use of exoskeleton technology in violin education, it is essential to assess it within a realistic simulation of its intended educational context^30^, with active involvement from the music education community throughout the development process^31^. Qualitative feedback from the learners reflects the embodied, sensorimotor, and expressive dimensions of practice^32^, while qualitative teacher assessments provide expert insight into technical accuracy, musicality, and pedagogical effectiveness^33^.

On the other hand, a quantitative framework for evaluating the educational benefits of haptic-assisted training^31^, as well as rehabilitation and motor re-learning^34^, remains underdeveloped. Such framework can be based on motion capture (MoCap)^33^, optical sensors^35^, infrared depth cameras^36^, or audio information retrieval techniques^36^, among others, and should focus on three key areas: spatial, temporal, and spatiotemporal metrics^11^.

In the context of violin playing, spatial metrics include measures such as joint range of motion (ROM)^37^, movement variability^38^, and interjoint coordination^38^. Temporal metrics assess timing aspects, such as delays in bowing changes between student and teacher^39^. Spatiotemporal metrics combine both dimensions, for example, by measuring the Euclidean distance between the bowing movements of teacher and student^40^, or by assessing movement smoothness during bowing^41–43^.

Therefore, in this work, we start from a multimodal approach that integrates both quantitative and qualitative markers to more effectively evaluate the technology in alignment with actual music practice and the literature on haptic-assisted learning. Subsequently, we propose a quantitative framework, in which a selection of quantitative metrics, derived from expert evaluations, is used to track learning progress over time. This approach bridges qualitative and quantitative dimensions of skill acquisition, thereby enabling a quantitative assessment of haptic-assisted training in music education.

### Study description

In this study, the effect of robotic exoskeleton-mediated motor learning on acquiring basic violin playing techniques is examined through haptic-assisted training (as proposed in^44^. Two groups of participants were assessed: one group wore a robotic haptic exoskeleton device throughout the experiment (*N* = 12), while the second group did not use any haptic device (*N* = 12) in a mixed between-within-subjects design.

All participants were novices at playing the violin. Their skill levels and general affinity for music were evaluated using questionnaires and a baseline measurement. The experiment began with a familiarization phase, followed by a baseline measurement consisting of three basic violin exercises. Participants then received a 20-minute prerecorded video lesson providing detailed instructions on how to perform the exercises, referred to as the training measurement. After a 10-minute break, all participants repeated the exercises in a recall measurement to evaluate short-term learning.

The group wearing the robotic haptic exoskeleton received real-time haptic feedback to guide correct movements of the right arm while holding the bow. Feedback was only provided during the lesson, not during baseline or recall measurements. The exoskeleton-assisted group is referred to hereafter as AVE (audio-video-exo), while the control group, which trained using only audiovisual learning materials, is referred to as AV (audio-video).

To measure violin playing technique during baseline and recall measurements, participants’ performances were evaluated qualitatively by an independent expert panel. In addition, participants completed self-assessments regarding their performance, effort, and the effectiveness of the lesson.

Motor learning outcomes were evaluated using a quantitative framework based on a selection of kinematic parameters.

Participants’ performance was evaluated during baseline, training and recall measurements. Since a retention measurement was not performed, only short-term motor learning was assessed (e.g.,^14,16,45^).

In addition, audio and video recordings were made for all measurements, and the exoskeleton’s torque and angle data were recorded; however, these additional data were not analyzed in the current study.

### Research hypotheses

This study investigates the educational potential of haptic-assisted training using a multimodal approach, combining qualitative and quantitative metrics. To ensure that quantitative measures meaningfully reflect violin performance, we first examine how they relate to expert evaluations:

- Hypothesis 1: Expert ratings of violin technique correlate with quantitative metrics.
  - Hypothesis 1.1: Ratings correlate with temporal metrics.
  - Hypothesis 1.2: Ratings correlate with spatial metrics.
  - Hypothesis 1.3: Ratings correlate with spatiotemporal metrics.

Haptic-assisted education is expected to support the learning process by enhancing the perception of correct bowing movements. Therefore, the second hypothesis is:

- Hypothesis 2: Haptic feedback leads to improved performance during training.
  - Hypothesis 2.1: This is reflected in the temporal metrics.
  - Hypothesis 2.2: This is reflected in the spatial metrics.
  - Hypothesis 2.3: This is reflected in the spatiotemporal metrics.

Finally, we anticipate that these benefits will translate into short-term retention of the skills. Therefore, the third hypothesis is:

- Hypothesis 3: Haptic feedback leads to improved recall measurements compared to the baseline.
  - Hypothesis 3.1: This is reflected in the expert ratings.
  - Hypothesis 3.2: This is reflected in the temporal metrics.
  - Hypothesis 3.3: This is reflected in the spatial metrics.
  - Hypothesis 3.4: This is reflected in the spatiotemporal metrics.

## Materials and methods

### Exoskeleton technology

#### Exoskeleton characteristics

**Fig. 1:**
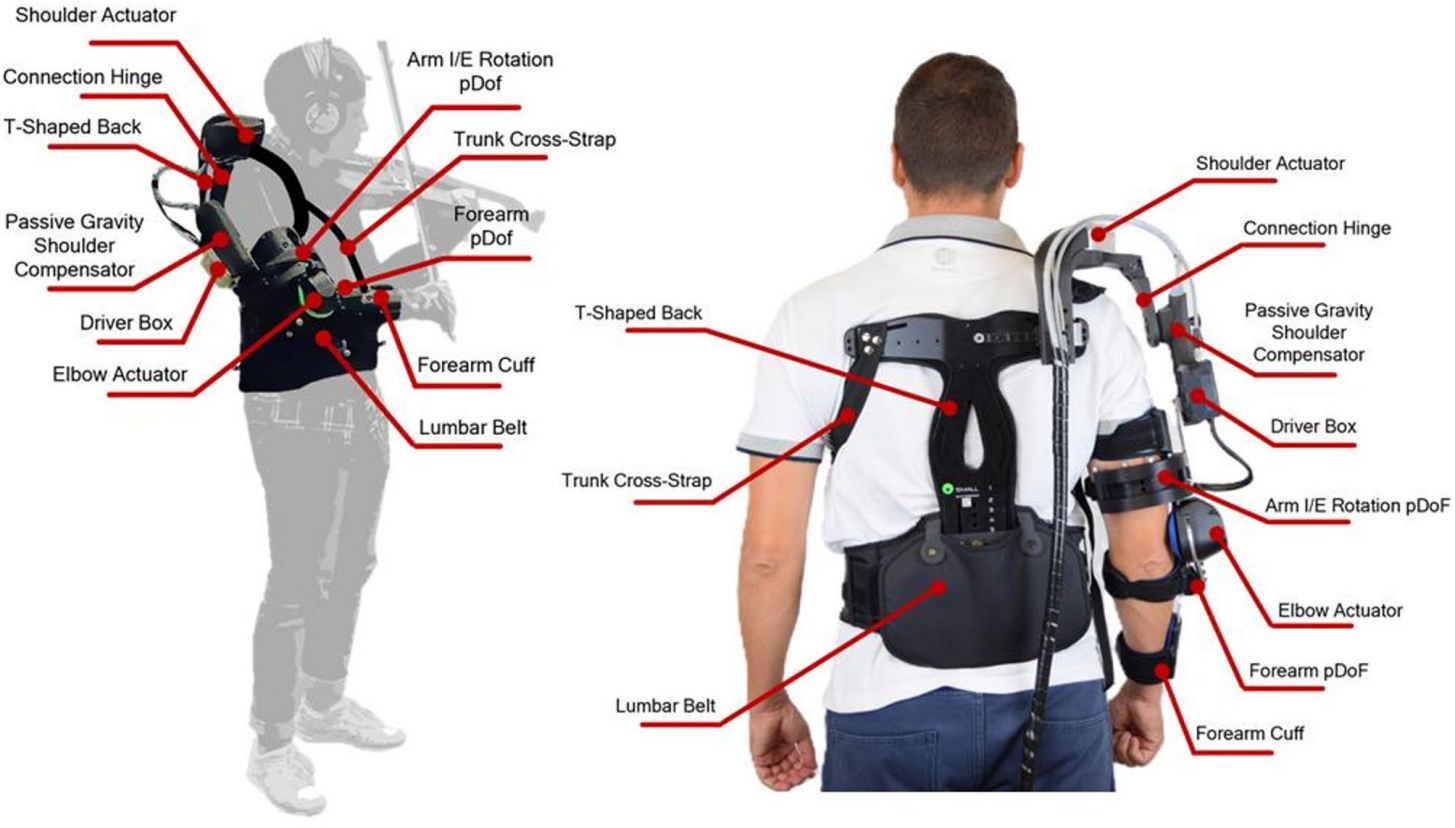
Two views of the exoskeleton, consisting of the shoulder module and the elbow module. The actuators and the passive unloader are hosted in 3D printed parts, connected to a carbon fiber T-shaped frame. The active components are connected through a lockable hinge anchored to the passive shoulder gravity compensator. The overall structure is attached to the user’s body through three main anchoring points: the semi-rigid lumbar belt, the trunk cross-strap and the arm and forearm cuffs, all using Velcro attachments to enable fast and easy donning/doffing and adaptability to different anthropometries. Finally, the passive internal/external (IE) rotation joint allows the physical connection of shoulder and elbow modules and the decoupling of their movements.

The exoskeleton used in this study was developed within the framework of the CONBOTS project (Grant 871803)^46^ and is depicted in Fig. 1. It is composed of a powered shoulder actuator, a passive shoulder gravity compensation system, a powered elbow actuator, and a physical human-robot interface (pHRI), all connected to remote electronics boards. The overall weight of the wearable device, since the electronics boxes are remotely located, is approximately 3.7 kg.

The shoulder part integrates both an active and a passive shoulder module. The active module comprises an actuated joint with a vertical rotation axis, actuating horizontal abduction/adduction (AA). The passive shoulder gravity compensation (PSGC) system is based on a spring-loaded mechanism that provides a bell-shaped torque profile supporting shoulder elevation^47^. The torque profile is set to counterbalance the weight of the more distal parts. Additionally, the PSGC is equipped with an absolute magnetic encoder to measure the elevation angle. A snap pin is used to engage or disengage the spring mechanism, enabling the assistance to be removed or set. The elbow module has the actuator axis aligned with the anatomical flexion/extension (FE) axis of the elbow^48^.

The exoskeleton incorporates a set of adjustments in its pHRI to fit the structure according to the user’s anthropometric dimensions, along with two passive degrees of freedom (pDoFs) to increase freedom of motion while wearing the rigid structure. It includes a custom carbon fiber T-shaped back frame connected to the user’s waist via a semi-rigid lumbar belt. The elbow module is integrated into a customized commercial elbow brace (Innovator X, Össur, Reykjavik, Iceland). Several belts and straps secure the device to the trunk, ensuring stability by counterbalancing its weight, which is mainly distributed on the right side.

An adjustable and lockable hinge, pivoting along the internal/external (IE) axis of the shoulder, is used to align the PSGC with the corresponding elevation (E) axis of the shoulder. A linear slider adjusts the relative position of the PSGC and the shoulder hinge, acting along the E axis of the user’s arm. Moreover, the exoskeleton is equipped with two pDoFs to guarantee unhindered motion during bowing movements. A passive IE rotation joint connects the PSGC to the elbow module, enabling forearm rotation around the humeral axis. For the elbow, the commercial brace has been modified to incorporate a rotational pDoF placed between the output link of the module and the forearm brace to accommodate pronation/supination (PS) movements of the wrist.

The system consists of two remote electronics boxes, one for each active module.

#### Exoskeleton control strategy

Two control strategies were embedded in the firmware for the control of the active modules during the exoskeleton operation, namely (i) *transparent mode* (TM), i.e. with a null output torque as the desired reference, and (ii) *haptic mode* (HM), in which a desired reference angular trajectory is defined for the elbow and shoulder joints based on a predefined equilibrium point or an angular trajectory stored in the exoskeleton’s memory.

Participants can record and play back a trajectory for the two active joints, thus performing a gesture, record the different trajectories in TM mode, and then use them as inputs to the mid-level control as a reference, acting in playback. The exoskeleton will then apply a haptic torque based on the current movement. The HM was implemented as a viscous-elastic force field acting at the shoulder and elbow level, which generates the desired torque according to the error between measured angle at the joint and the desired trajectory recorded in the exoskeleton memory. This control layer is implemented in the real-time processor of the exoskeleton running at 100 Hz.

These two control modalities have been employed with two different aims. Specifically, the TM was used during the baseline and recall phases of the experimental protocol to evaluate the pre- and post-training performance of the users; under this experimental condition, the exoskeleton provided neither assistance nor resistance to the users, just being used as a measurement tool to record human trajectories while playing. Conversely, the HM was used during the training phase, where haptic feedback is needed to enable the robot-assisted training.

### Audiovisual material

#### Training video

**Fig. 2:**
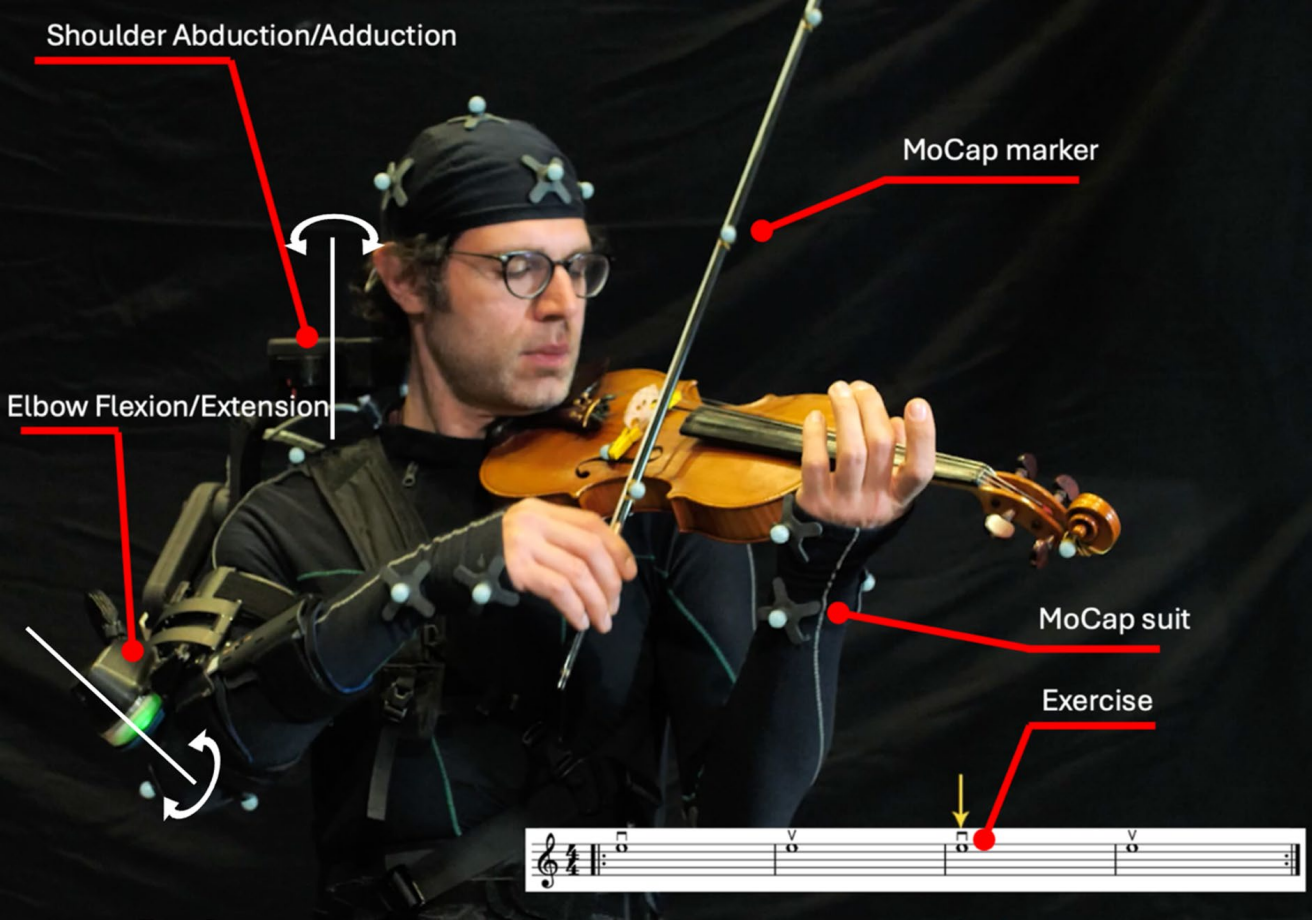
Screenshot from the training video. The teacher’s playing was shown from 2 angles, alternating throughout the exercise. In the meanwhile, the exercise was displayed in the lower right corner as a reference. The teacher is wearing a MoCap suit and the exoskeleton, covered in MoCap markers, adhering as closely as possible to the Animation Marker Set configuration. The haptic actuators of the exoskeleton of shoulder and elbow are indicated on the Figure, with their respective axes of rotation.

**Fig. 3:**
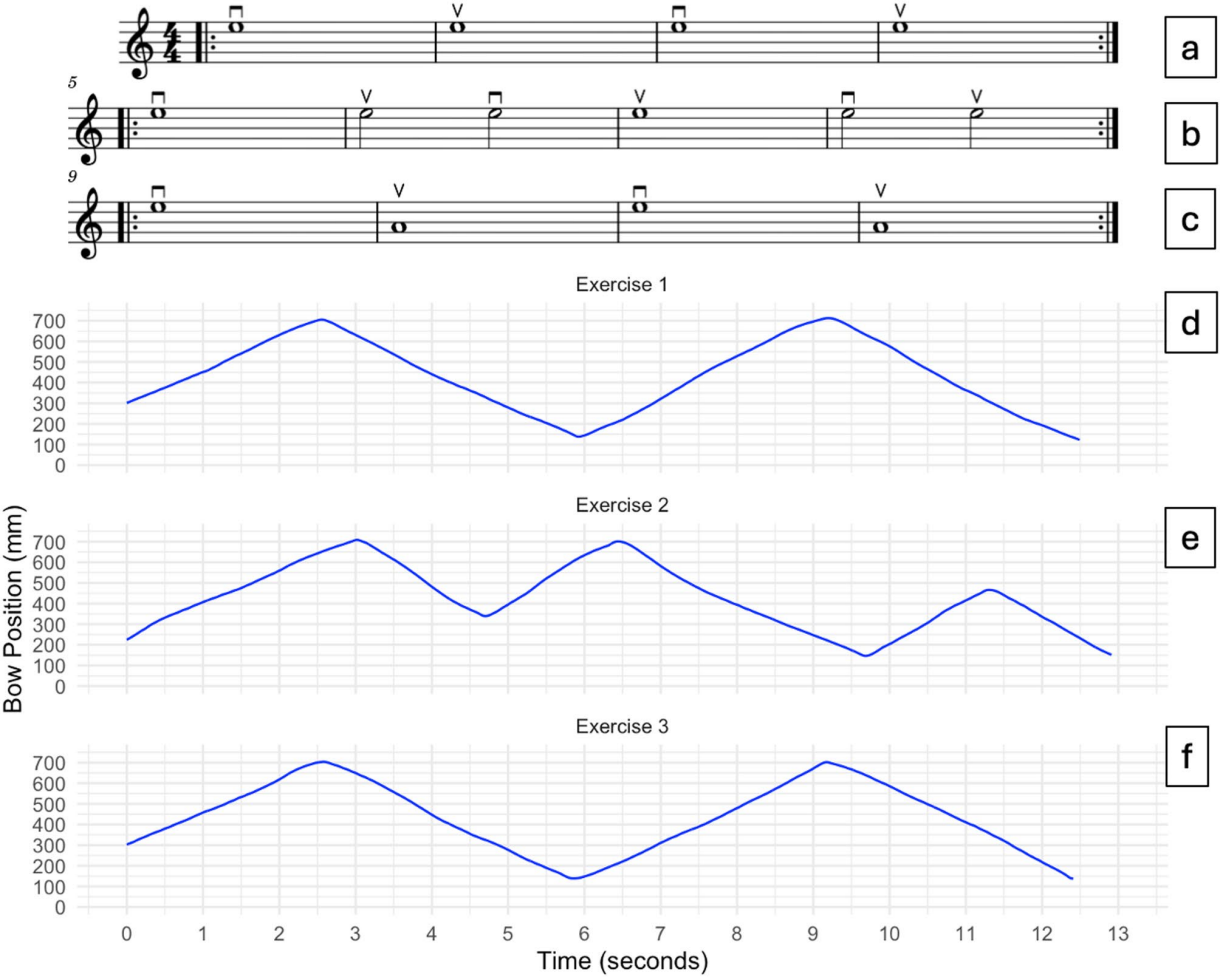
The three different exercises used in the protocol. Exercise 1 (**a**), 2 (**b**) and 3 (**c**) are to be played with the bowing pattern as indicated by the bow markings. The respective corresponding bow positions of the teacher are shown in (**d**), (**e**) and (**f**).

**Fig. 4:**
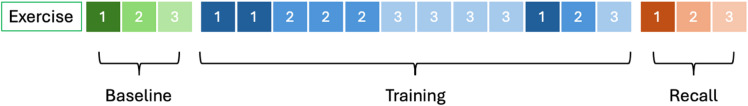
an overview of the order of execution of the 3 exercises throughout the experiment, during baseline (green), training (blue) and recall (red) measurements.

A meticulously scripted training video, narrated by an expert violinist and teacher, was recorded for the experiment (see Fig. 2, for the entire video: see Supplementary Materials). The 20-minute video, instructs correct execution of three basic violin exercises (exercise 1, 2, and 3, see Fig. 3, for the videos of the exercises: see Supplementary Materials), and it follows a structured format, explaining and reiterating the three exercises throughout the video (see Fig. 4):

- Introduction covering the violin and bow.
- Preparation of exercise 1: Full bows down and up on the G-string (4x, Fig. 3.a).
- Joint performance of exercise 1 (2x in total).
- Preparation of exercise 2: A more intricate bowing pattern involving full bow down, half bow up, half bow down, full bow up, half bow down, half bow up (2x, Fig. 3.b).
- Joint performance of exercise 2 (3x in total).
- Preparation of exercise 3: Full bows down and up, alternating between the G and D strings (4x, Fig. 3.c).
- Joint performance of exercise 3 (4x in total).
- General recapitulation.
- Joint performance of exercise 1 (1x in total).
- Joint performance of exercise 2 (1x in total).
- Joint performance of exercise 3 (1x in total).

**Fig. 5:**
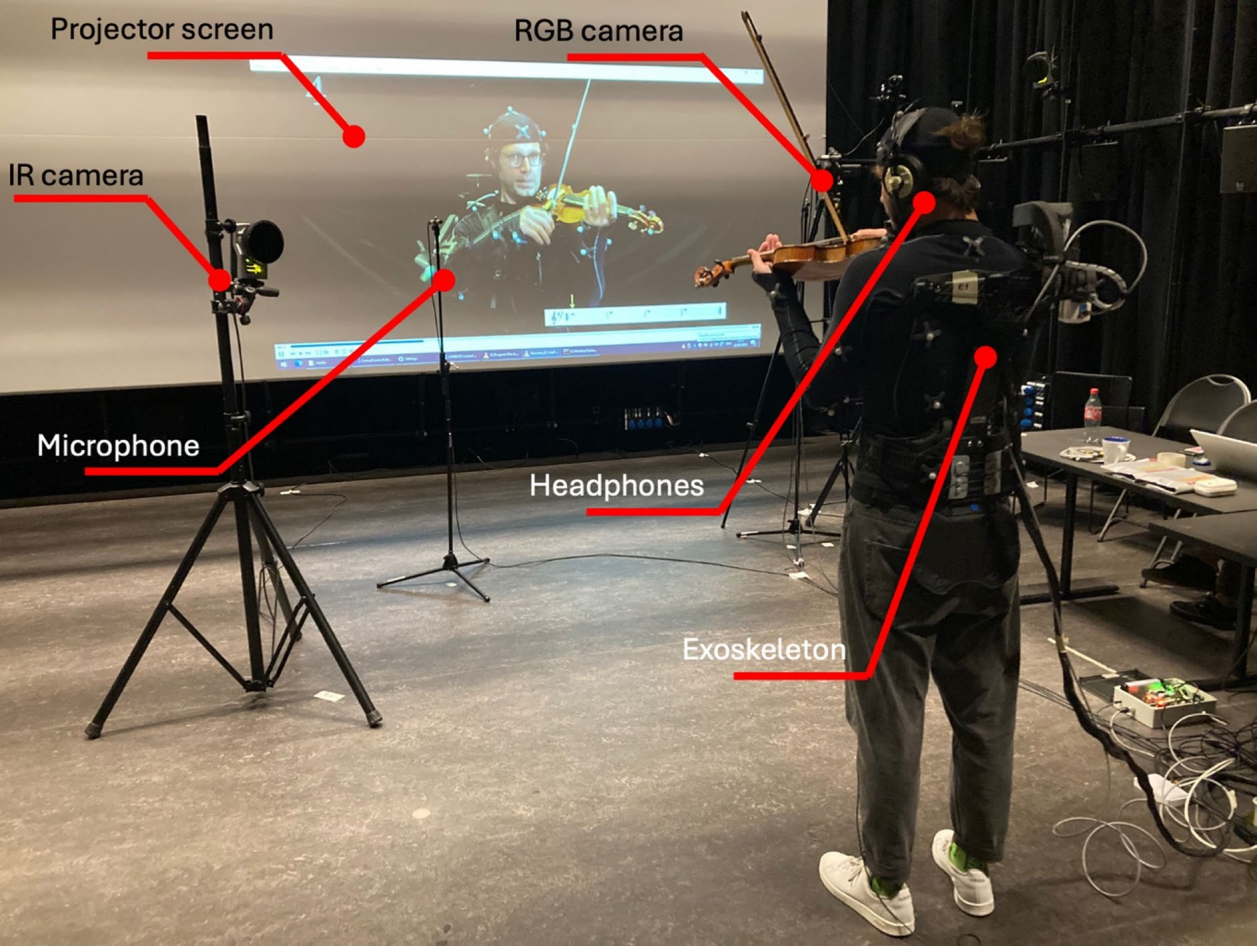
Experimental setup, featuring an AVE participant. The projector screen displays the teacher wearing the exoskeleton, and a MoCap suit. The participant is wearing an exoskeleton and MoCap suit as well. Motion is captured by IR MoCap cameras, and an RGB camera makes a recording of participants’ performance. In addition, 2 microphones record the audio. The participant faces the teacher on the screen and plays the violin in synchronization with the teacher. The audio of the teacher is delivered through headphones.

A depiction of the entire measurement setup, featuring an AVE participant, can be seen in Fig. 5. The video angle alternates throughout the video, to enhance demonstration of correct violin technique where necessary.

#### Baseline and recall videos

Additionally, separate recordings of exercise 1, 2, and 3 were created by the same teacher, analogous to the training video. These recordings will be used eventually to acquire baseline and recall measurements from the participants. In all videos from the preparation step, the recordings integrate kinematic data from the exoskeleton, video, audio, and MoCap data. The synchronization of all data streams was achieved using the SMPTE protocol. An overview of the sequence of exercises 1, 2, and 3 throughout the experiment, combining baseline, training and recall measurements, is shown in Fig. 4.

### Measurement protocol

#### Ethical approval

The experiment received ethical approval from the UGent ethical committee (ref. 2022-38). Informed consent was obtained from all participants. All methods were performed in accordance with the relevant guidelines and regulations of the involved institutions, and in accordance with the Declaration of Helsinki. Informed consent was obtained from the author/individual for publishing the image in Figs. 2 and 5, and for the videos and files in the Supplementary Materials.

#### Participants

In total, 24 participants were recruited (see Table 1). Inclusion criteria required participants to be male, approximately the same size as the teacher (see Table 1), and right-handed, ensuring anthropometric parameters closely matched those of the teacher. In addition, participants were required to be novices in violin playing.

Consequently, 12 participants underwent training with the wearable exoskeleton as described above (AVE), while a control group of 12 participants trained and were tested under identical conditions without the exoskeleton (AV).

**Table 1:** Overview of the participants’ characteristics.

AVE (*N* = 12)					AV (*N* = 12)				
Participant number	Age (years)	Weight (kg)	Length (cm)	MSI (a.u.)	Participant number	Age (years)	Weight (kg)	Length (cm)	MSI (a.u.)
1	38	82	185	4.4	13	28	70	175	4.0
2	63	80	192	5.4	14	21	70	185	4.2
3	39	80	197	4.9	15	36	72	182	3.3
4	35	64	184	3.1	16	40	70	180	4.6
5	28	67	169	4.4	17	38	95	186	2.9
6	40	85	185	4.3	18	29	81	185	4.6
7	42	71	181	3.3	19	25	61	176	3.9
8	40	75	183	4.1	20	30	75	178	3.3
9	22	73	175	3.3	21	31	85	194	3.9
10	50	95	178	4.8	22	53	75	184	4.3
11	23	75	183	2.9	23	28	84	198	5.1
12	27	85	187	3.8	24	45	79	185	4.2
median	39	78	183.5	4.2	median	31	75	184.5	4.1
IQR	14	13	7.8	1.4	IQR	12	13	7.3	1.1
Teacher	42	80	181						

AV = control group, AVE = haptic group, IQR = interquartile range, MSI = Music Sophistication Index.

#### Experimental workflow

**Fig. 6:**
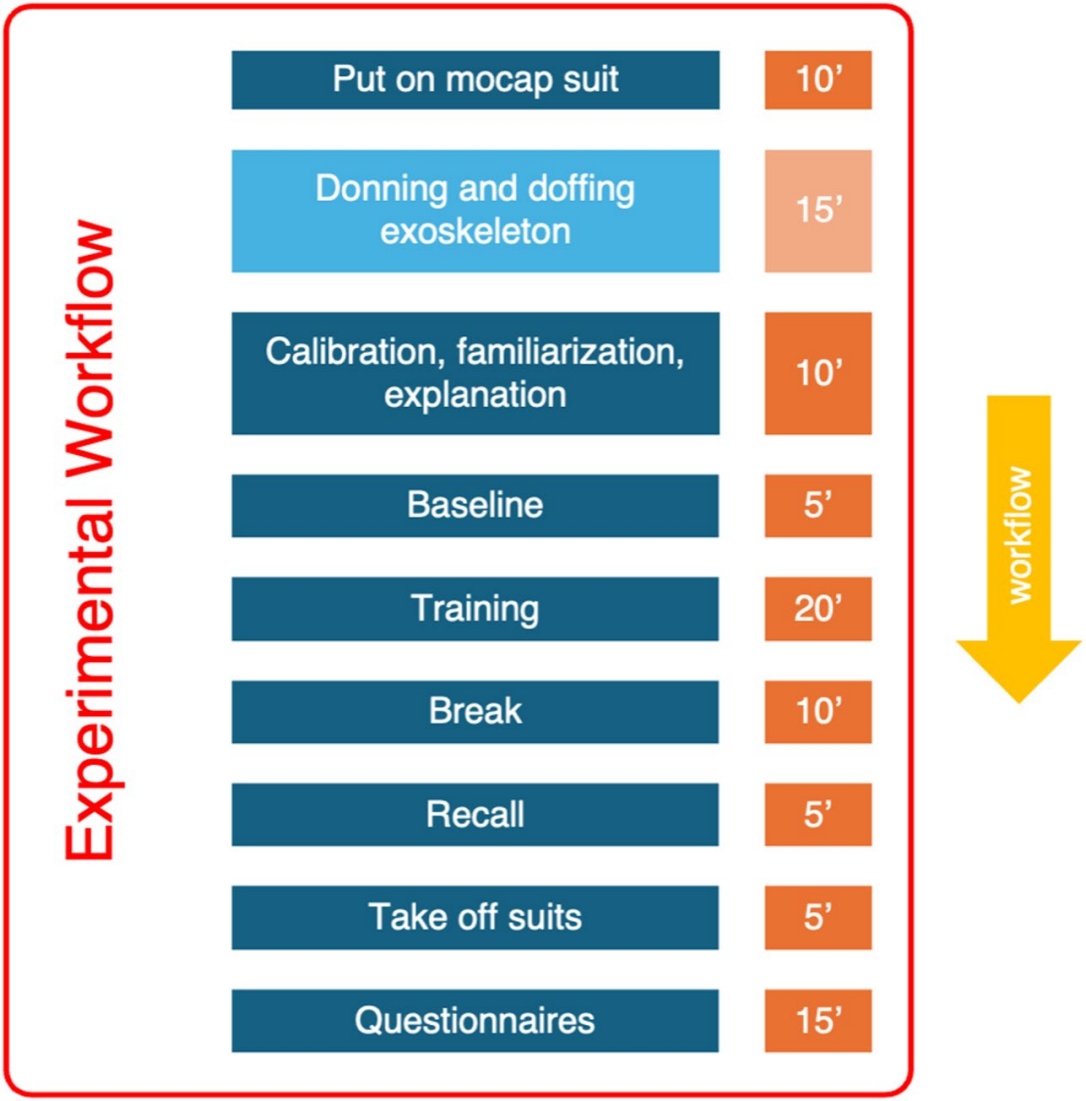
Overview of the experimental workflow, following the preparation of the audiovisual material. The box in lighter blue is only performed by AVE. The orange boxes indicate duration of each action.

The experimental workflow following the preparation of the audiovisual material is depicted in Fig. 6.

##### Familiarization phase and baseline measurement

First, participants familiarized themselves with the exoskeleton, and the MoCap model was calibrated. Following this phase, participants received a brief primer on holding the violin and bow, and the experimental procedure was explained. They were instructed to replicate the movements demonstrated in the training video and to copy all movements of the teacher in sync wherever possible.

During the baseline measurement, participants viewed videos of exercises 1, 2, and 3, projected on a large screen while standing in the MoCap area. The exoskeleton was set to TM in the AVE condition during these exercises.

##### Training

After the baseline measurement, participants watched the training video, again explicitly instructed to copy all movements of the teacher in sync wherever possible. In the AVE condition, the exoskeleton was switched to HM (see Fig. 6), providing feedback on the position of the elbow and shoulder via a viscous-elastic force field. This guided correct movements of the right arm by providing concurrent haptic torque feedback on the flexion-extension (FE) of the right elbow and the horizontal abduction-adduction (AA) of the right shoulder, aimed at enhancing participants’ performance.

##### Recall measurement and questionnaires

After the training and a short break, participants repeated the three exercises during a recall measurement. During this measurement, participants again viewed the videos for exercises 1, 2, and 3 and were explicitly instructed to copy all movements of the teacher in sync wherever possible. In the AVE condition, the exoskeleton control was switched back to TM.

Importantly, due to practical considerations, a retention measurement was not included in the study; thus, only short-term learning, whether actual motor learning or motor adaptation, was measured.

Finally, participants completed the Music Sophistication Index (MSI) questionnaire^49^, a user experience questionnaire, several questions related to their background in violin playing, and a self-assessment questionnaire.

### Data acquisition

#### Expert panel

**Fig. 7:**
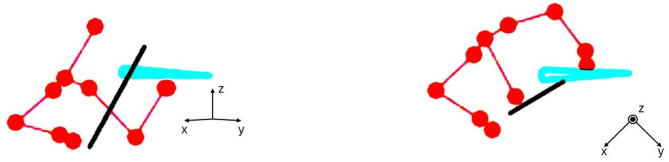
stick-figure rendition of the MoCap data. In the same video, a synchronized sideways and an upside-down view was included, to facilitate evaluation of bowing technique. The red dots and lines represent the upper body of the participant, the black line is the violin bow, the blue triangle is the violin.

All participants’ exercises as performed during baseline and recall measurements, were evaluated by a panel of five double-blind violin experts. The experts were all professional violinists, with at least 30 years of playing experience. The evaluation was conducted on anonymized stick-figure videos of the upper body, generated from the MoCap data streams (see Fig. 7). Furthermore, the use of stick-figures prevented the identification of whether participants were wearing an exoskeleton and whether the video was taken before or after the training measurement. Each video depicted two perspectives of the player simultaneously. The experts evaluated the movies on a Likert scale ranging from 1 to 7, where 1 represented “very bad” and 7 represented “very good.” Each expert rated all videos pertaining to a specific exercise sequentially, starting with exercise 1, followed by exercise 2, and concluding with exercise 3. The movies were played without sound to ensure that experts focused solely on evaluating technique, free from any influence of audio cues. In total, there were 144 videos, which were per exercise presented in randomized order.

#### Questionnaires

After training and recall measurements, participants in AV and AVE completed 2 and 3 questionnaires, respectively. The first questionnaire evaluated their musical background using the standardized MSI questionnaire. The second questionnaire was a self-evaluation regarding participants’ perceptions of their own performance during baseline and recall measurements, the perceived difficulty of the exercises, and the perceived effectiveness of the training in performing exercises 1, 2, or 3. The second questionnaire concluded with open questions about the training structure and potential improvements for future iterations. The final questionnaire focused on AVE, exploring their overall experience with the exoskeleton. It consisted of open questions and a series of questions requiring responses on a Likert scale (ranging from 1 to 5, with 1 representing complete disagreement and 5 representing complete agreement). Copies of the questionnaires can be found in the Supplementary Materials.

#### Multimodal data acquisition

To reliably and reproducibly measure and compare the movements of participants and instructors, a Qualisys MoCap system (Qualisys AB, Gothenburg, Sweden) was employed. This system uses multiple high-speed infrared cameras to track reflective markers placed on key anatomical landmarks. Participants wore an upper-body MoCap suit equipped with 24 reflective markers (see Fig. 2), following the Qualisys Animation Marker Set configuration^50^, with slight adjustments to marker positions to maintain consistency between the AV and AVE groups. Additionally, four markers were affixed to the violin and three to the bow (Fig. 2) to measure bow positions relative to the instrument^40^.

The system in this study utilized 18 infrared cameras, strategically positioned to ensure comprehensive coverage from all angles and to prevent gaps in data capture.

MoCap data were recorded at 120 Hz, while audio was recorded using a Y-pair of condenser microphones at 48 kHz with 24-bit depth. An RGB camera faced the participant to provide visual recordings.

Audio, video, and MoCap data were recorded simultaneously and synchronized post hoc using the Society of Motion Picture and Television Engineers (SMPTE) standard to label all data streams with a unique timestamp.

The instruction video was played back on the screen and through headphones. In the AVE group, synchronization between video, audio, and exoskeleton feedback was achieved through the Central Control Software (CCS), specifically developed for the CONBOTS project^51^.

Multimodal recordings were obtained during the baseline, training, and recall measurements.

### Data analysis

#### Motion capture data and performance metrics

**Fig. 8:**
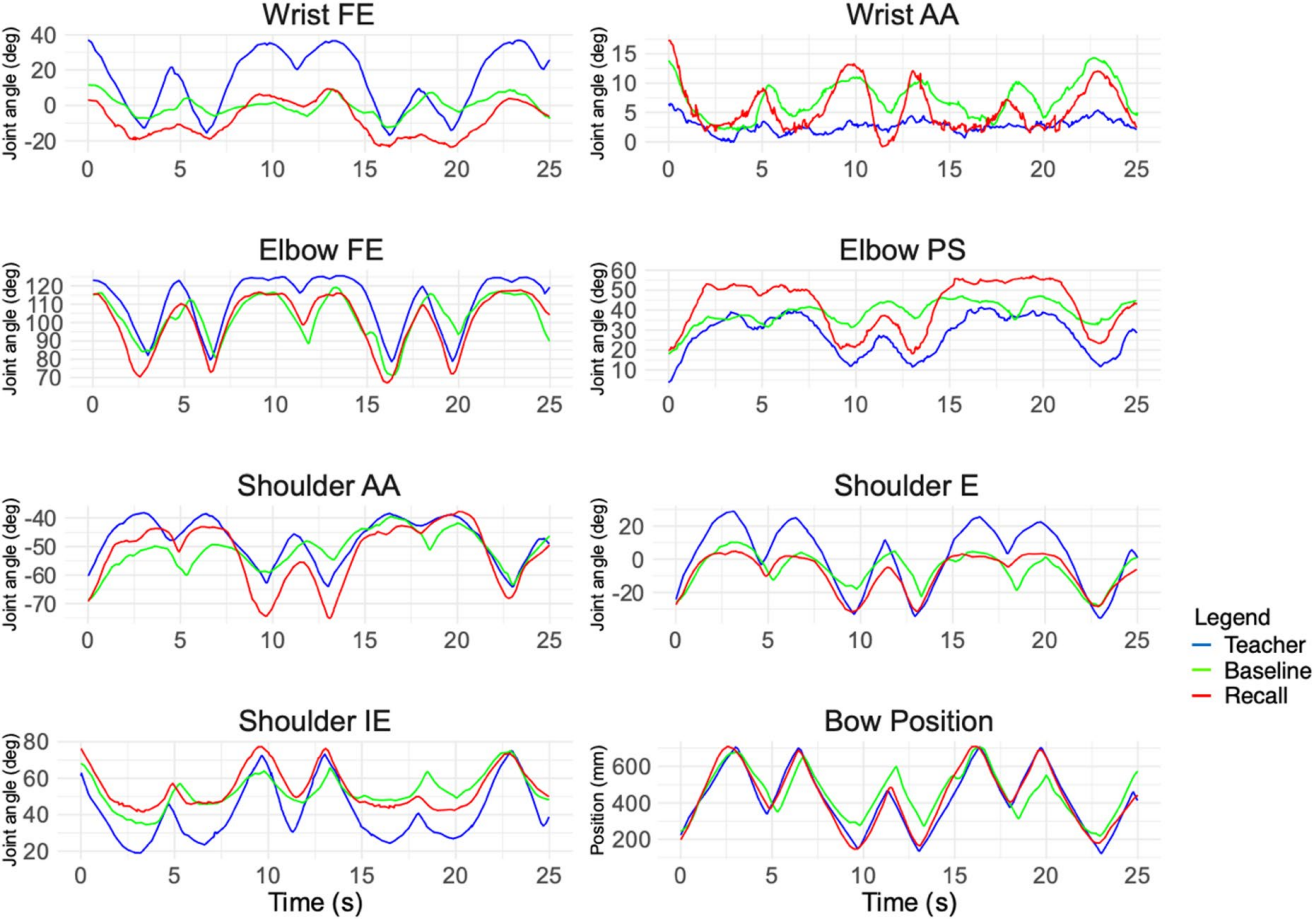
joint angles of the upper body were calculated, and bow position relative to the bridge and strings. Here the performance of a participant in exercise 2 is shown during baseline (green) and recall (red) measurements. The example as given by the teacher is given in blue. Angles of wrist (flexion/extension (FE), adduction/abduction (AA)), elbow (FE, pronation/supination (PS)) and shoulder (AA), elevation (E), internal/external rotation (IE)) are given, as well as the bow position relative to the string.

Joint angles of wrist, elbow and shoulder were approximated from the MoCap data using a custom-made MATLAB (MATLAB version R2023, MathWorks, Natick, MA, USA) package^40,52,53^, based on the standards of the ISB^54–56^ In addition, angles and positions of bow and violin were calculated (see Fig. 8).

The comparison between teacher and participant data involved an assessment of each analyzed degree of freedom (DoF), including horizontal AA, elevation (E), and IE rotation of the shoulder, as well as FE and PS of the elbow, and finally, FE and AA of the wrist joints. Additionally, the bow position relative to the contact point with the string was considered.

Additionally, from the analyzed MoCap data, a set of 17 metrics was derived, based on the literature, advice of violin experts, and professional experience from involved researchers. These performance metrics were computed separately for baseline and recall measurements, as well as for the training measurement. The metrics were subdivided into the following arbitrary classes:

- Spatial performance metrics that evaluate, for example, the ROM (e.g.,^57^), interjoint coordination (e.g.,^58,59^), or variability (standard deviation of errors, e.g^38,60,61^) . The spatial metrics used in this study are:
  - Metric 1: The full range of the bow used throughout the exercise
  - Metric 2: The ROM of elbow FE used throughout the exercise
  - Metric 3: The ROM of shoulder AA used throughout the exercise
  - Metric 4: The interjoint coordination of elbow FE and shoulder AA, defined as the ratio of the ROM of elbow FE to the ROM of shoulder AA in the upper half of the bow
  - Metric 5: The interjoint coordination of elbow FE and shoulder AA, defined as the ratio of the ROM of elbow FE to the ROM of shoulder AA in the lower half of the bow
  - Metric 6: The coordination between shoulder AA and bow, defined as the ratio of the ROM of shoulder AA to the portion of the bow used in the upper half of the bow
  - Metric 7: The coordination between shoulder AA and bow, defined as the ratio of the ROM of shoulder AA to the portion of the bow used in the lower half of the bow
  - Metric 8: The coordination between elbow FE and bow, defined as the ratio of ROM of elbow FE to the portion of the bow used in the upper half of the bow
  - Metric 9: The coordination between shoulder and bow, defined as the ratio of ROM of elbow FE to the portion of the bow used in the lower half of the bow
  - Metric 10: The variability in elbow FE when performing a bowing movement
  - Metric 11: The variability in shoulder AA when performing a bowing movement
  - Metric 12: The variability in the interjoint coordination between elbow FE and shoulder AA when performing a bowing movement

- Temporal performance metrics that evaluate aspects of timing, such as rhythm and synchronicity (e.g., ^16^,^62^). The temporal metrics used in this study are:
  - Metric 13: Timing difference between participant and teacher, defined as the difference in the changes of direction in bowing between student and teacher

- Spatiotemporal performance metrics that measure, for example, the Euclidean difference between a trajectory and a reference trajectory (deviation from reference trajectory in task and joint space, e.g.,^16,29,63^), or movement smoothness (spectral arc length or SPARC, e.g., ^64^), the latter of which might indicate physical and mental effort^65^. The spatiotemporal metrics used in this study are:
  - Metric 14: The Euclidean distance of bow position between participant and teacher
  - Metric 15: SPARC, or movement smoothness
  - Metric 16: Synchronization strength, consistency in timing difference between participant and teacher
  - Metric 17: Phase shift, or difference in timing between participant and teacher

#### Statistical analysis

##### Questionnaires

Self-evaluation, perceived effectiveness of the training, and perceived difficulty of the exercises were investigated using the brms package in R, using a cumulative distribution, using the logit function as link function. The primary effect considered was Group (factor 1,2). Random effects encompassed the interaction between Participant (factor 1–24) and Exercise (factor 1–3). The model in R syntax is for the 3 cases defined as follows:

$$\:\mathrm{R}\mathrm{a}\mathrm{t}\mathrm{i}\mathrm{n}\mathrm{g}\:\sim\:\mathrm{G}\mathrm{r}\mathrm{o}\mathrm{u}\mathrm{p}\:+\:\left(1\right|\mathrm{P}\mathrm{a}\mathrm{r}\mathrm{t}\mathrm{i}\mathrm{c}\mathrm{i}\mathrm{p}\mathrm{a}\mathrm{n}\mathrm{t}:\mathrm{E}\mathrm{x}\mathrm{e}\mathrm{r}\mathrm{c}\mathrm{i}\mathrm{s}\mathrm{e})$$

##### Metric correlations

17 metrics were correlated individually with mean expert ratings, using a Spearman correlation. A correction for multiple comparisons was applied, resulting in a significance threshold of *p* < 0.003. The results are shown in Table 2.

##### Motion capture data

The metrics that correlated significantly with expert ratings were retained for further analysis, supplemented with 6 other metrics (see Table 2 for the full list of metrics). These metrics were analyzed using the brms package in R. The primary effects considered were Group (factor 1,2), baseline/training/recall measurements (termed Stage, factor 1,2,3), and their interaction. Random effects encompassed the interaction between Participant (factor 1–24) and Exercise (factor 1–3).

The model formula in brms syntax was:

$$\:\mathrm{m}\mathrm{v}\mathrm{b}\mathrm{i}\mathrm{n}\mathrm{d}\left(\mathrm{S}\mathrm{e}\mathrm{l}\mathrm{e}\mathrm{c}\mathrm{t}\mathrm{e}\mathrm{d}\:\mathrm{M}\mathrm{e}\mathrm{t}\mathrm{r}\mathrm{i}\mathrm{c}\mathrm{s}\right)\:\sim\:0\:+\:\mathrm{G}\mathrm{r}\mathrm{o}\mathrm{u}\mathrm{p}\:\mathrm{*}\:\mathrm{S}\mathrm{t}\mathrm{a}\mathrm{g}\mathrm{e}\:+\:(1\:+\:\mathrm{S}\mathrm{t}\mathrm{a}\mathrm{g}\mathrm{e}\:|\mathrm{p}|\:\mathrm{P}\mathrm{a}\mathrm{r}\mathrm{t}\mathrm{i}\mathrm{c}\mathrm{i}\mathrm{p}\mathrm{a}\mathrm{n}\mathrm{t}:\mathrm{E}\mathrm{x}\mathrm{e}\mathrm{r}\mathrm{c}\mathrm{i}\mathrm{s}\mathrm{e})$$

Response variables were modeled as a skewed normal distribution. The model was run with 8000 iterations, and a warmup of 4000.

## Results

### Expert panel

Expert ratings (a Likert scale, with 1 = very bad, 7 = very good) improve significantly between baseline and recall qualitative measurements, in both groups, with an average improvement of 2.5 (highest posterior density (HPD): [2.0, 2.9]) in AV, and an average improvement of 3.1 (HPD: [2.6, 3.5]) in AVE. There is also a significant Group: Stage effect, with a relative improvement over Stage of 0.611 (HPD: [0.094, 1.232]) in AVE relative to AV. There was no significant difference in expert ratings between groups during baseline measurements. In conclusion, the findings suggest that Stage, as well as the interaction between Group and Stage, significantly influenced expert ratings. An overview of the contrasts from the expert panel’s statistical analysis is summarized in Table 3.

Inter-rater reliability was excellent, with a two-way random-effects absolute agreement intraclass correlation coefficient (ICC) of 0.71 for single raters (ICC(2,1), 95% CI [0.65, 0.77]) and 0.93 for the average of five raters (ICC(2,5), 95% CI [0.90, 0.94]).

### Motion capture data

#### Metric selection and correlation analysis

**Fig. 9:**
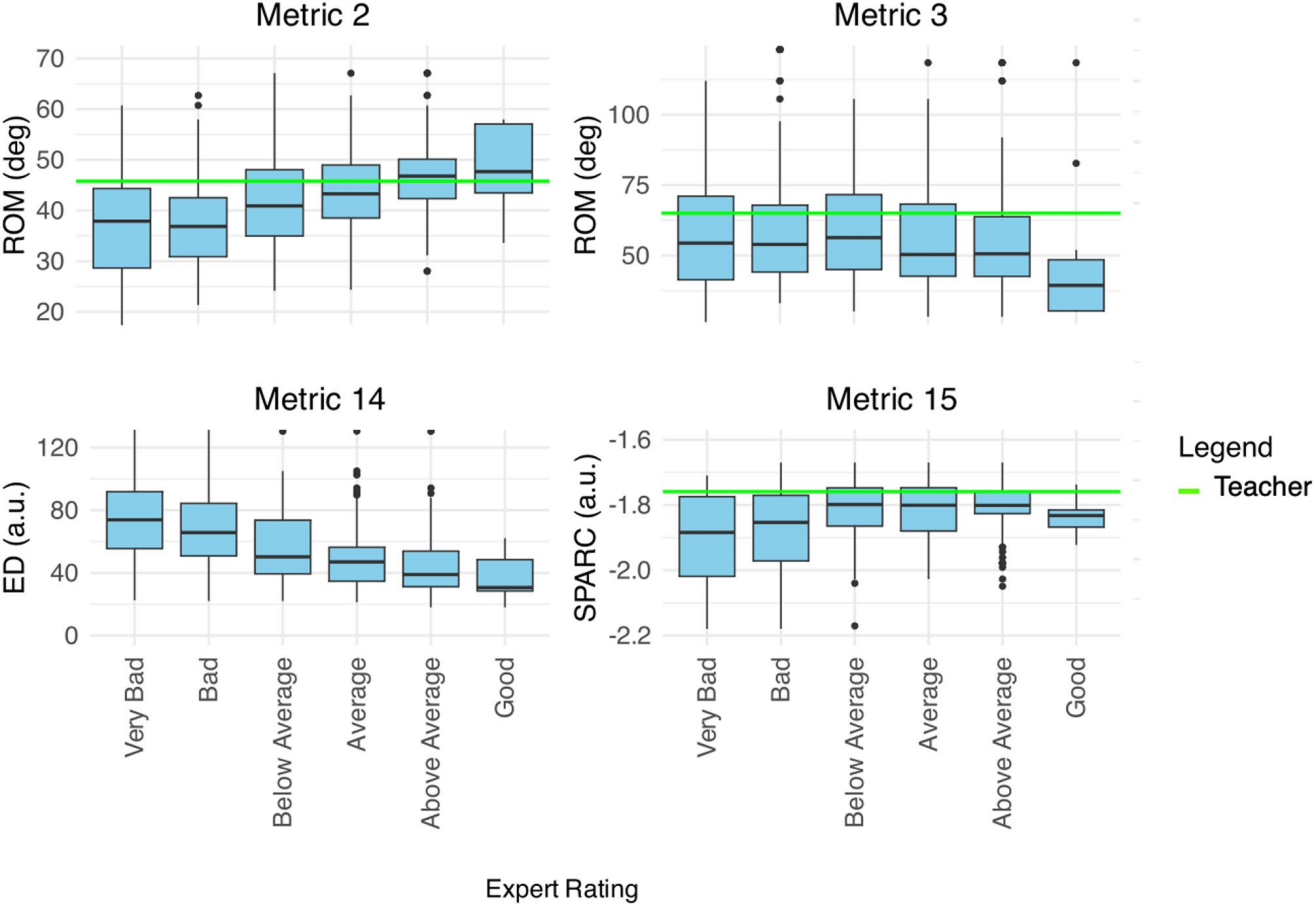
Several metrics exhibit significant correlation with expert ratings, which ranges from 1 (very bad) to 7 (very good). Notably, as participants demonstrate greater fidelity to the reference trajectory, indicated by the reduction in Euclidean distance (ED) of the bow movement (Metric 14), their expert ratings improve. Moreover, higher expert ratings correlate with an increase in the range of motion (ROM) exhibited by the elbow flexion/extension (FE, Metric 2), while they do not correlate with the ROM of the shoulder adduction/abduction (AA, Metric 3). Furthermore, participants’ spectral arc length (SPARC, Metric 15) showed significant correlation with expert ratings.

7 metrics showed significant correlation with expert panel outcome and were retained for further analysis. Additionally, a 6 metrics that did not correlate with expert ratings were included in the analysis for completeness (see Fig. 9).

The metrics that showed significant correlation with expert ratings were Metric 2, 4, 6, 8, 14, 15 and 16. The metrics that did not correlate significantly with expert ratings were Metric 1, 3, 5, 7, 9, 10, 11, 12, 13 and 17. A complete overview of the metrics and the results of the correlation analysis are provided in Table 2.

**Table 2:** Overview of the Spearman correlation of the 17 metrics involved in this study with expert ratings. Metrics that showed a significant correlation with expert ratings are indicated in bold; metrics that are included in subsequent analyses are indicated in italic. R and corrected p-values are given for each metric. Spatial metrics (Metrics 1–12), Temporal metrics (Metric 13), Spatiotemporal metrics (Metrics 14–17). AA = adduction/abduction, FE = flexion/extension, ROM = range of motion, SPARC = spectral arc length.

Metric number	Short description	Spearman *R*	*p*-value
1	full range bow used	0.24	0.0039
***2***	***ROM of elbow FE***	***0.42***	***<<<0.003***
*3*	*ROM of shoulder AA*	*-0.10*	*0.23*
***4***	***Interjoint coordination***,*** upper part bow***	***0.36***	***<<<0.003***
5	interjoint coordination, lower part now	0.14	0.098
***6***	***shoulder-bow coordination***,*** upper part bow***	***0.30***	***< 0.003***
7	shoulder-bow coordination, lower part bow	0.16	0.057
***8***	***Elbow-bow coordination***,*** upper part bow***	***-0.44***	***<<<0.003***
9	Elbow-bow coordination, lower part bow	-0.11	0.19
*10*	*variability elbow*	*-0.13*	*0.14*
*11*	*variability shoulder*	*-0.18*	*0.031*
*12*	*variability elbow-shoulder coordination*	*-0.21*	*0.013*
*13*	*timing*	*-0.095*	*0.26*
***14***	***Euclidian distance bow use***	***-0.55***	***<<<0.003***
***15***	***SPARC***	***0.28***	***< 0.003***
***16***	***Synchronization strength***	***-0.44***	***<<<0.003***
*17*	*Phase shift*	*0.037*	*0.66*

**Table 3:** Contrasts of expert panel assessment of the violin performances during baseline and recall measurements. Ratings of the expert panel were set between 1 (very bad) and 7 (very good).

Contrast	estimate	HPD.Lower	HPD.Upper
AV S1 - AVE S1	-0.6	-1.4	0.2
AV S1 - AV S3	-2.5	-2.9	-2.0
AVE S1 - AVE S3	-3.1	-3.5	-2.6
AV S3- AVE S3	-1.2	-2.0	-0.4
(AV S1 - AV S3) - (AVE S1 - AVE S3)	0.611	0.094	1.232

AV = control group, AVE = haptic group, HPD = highest posterior density, S1 = baseline measurement, S3 = recall measurement.

#### Metrics

In total, 13 metrics were retained for further analysis, as indicated in Table 2. In addition to the 7 metrics that showed significant correlation with expert ratings, to integrate this work with existing and future literature, and to evaluate the overall effect of the exoskeleton on upper limb kinematics, 6 extra metrics were further analyzed and discussed:

1. It must be noted that purely temporal metrics did not correlate with expert ratings. However, to measure the temporal effects of the exoskeleton technology, Metric 13 was retained for further analysis.
2. Given the active shoulder module in the exoskeleton, ROM of the shoulder AA in the analysis was added (Metric 3).
3. To integrate this work with previous work on sensorimotor synchronization, and future work on the evaluation of the used exoskeleton technology, the phase shift was also included in the analysis (Metric 17).
4. To evaluate the effect of the exoskeleton on variability during motor learning, Metrics 10, 11 and 12 were added.

**Fig. 10:**
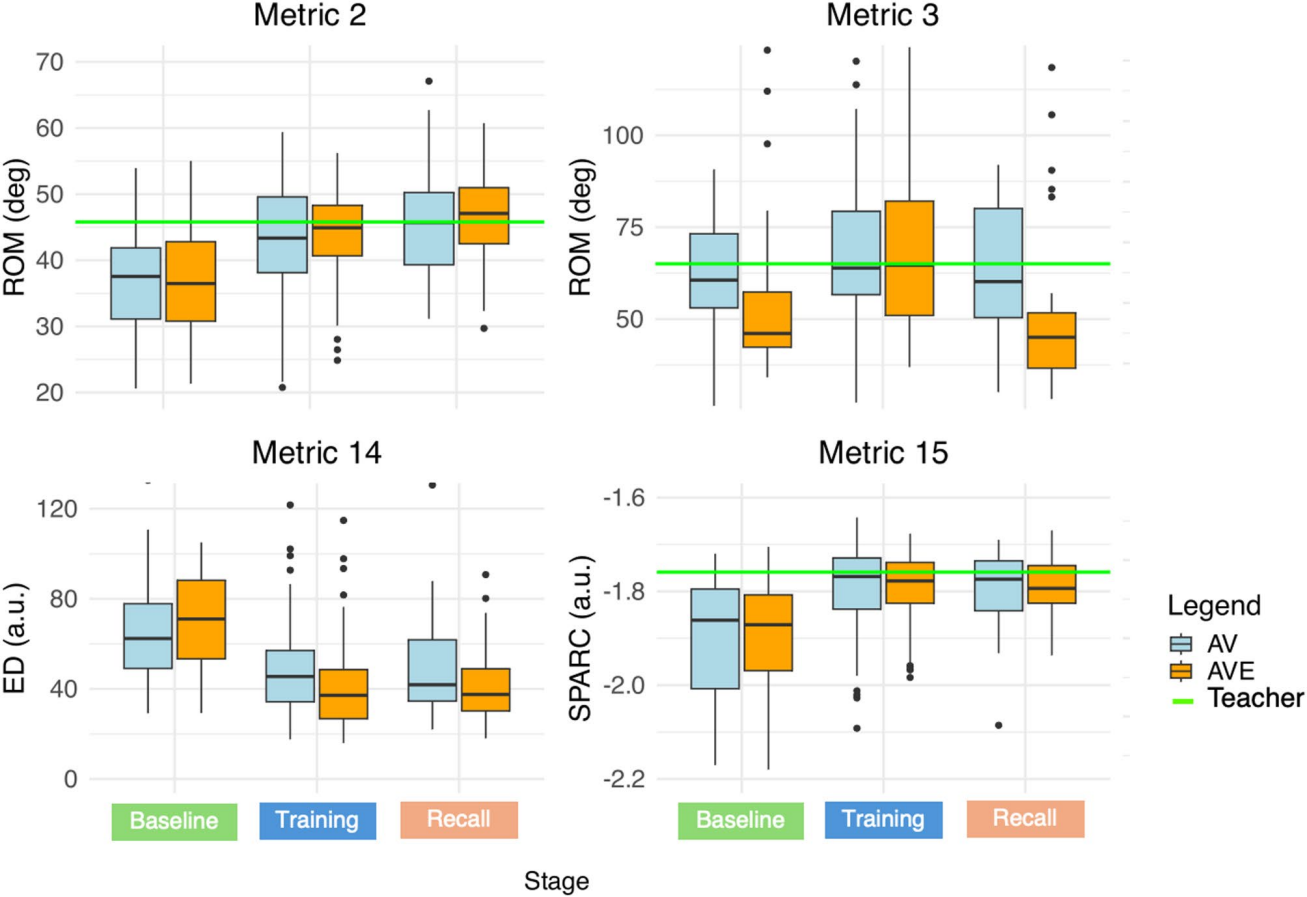
Metric 2, 3, 14 and 15 in AV and AVE, during baseline, training and recall measurements. The green line is the median performance of the teacher throughout the entire experiment. a.u. = arbitrary units, AV = control group, AVE = haptic group, deg = degrees, ED = Euclidian distance, ROM = range of motion, SPARC = spectral arc length.

**Fig. 11:**
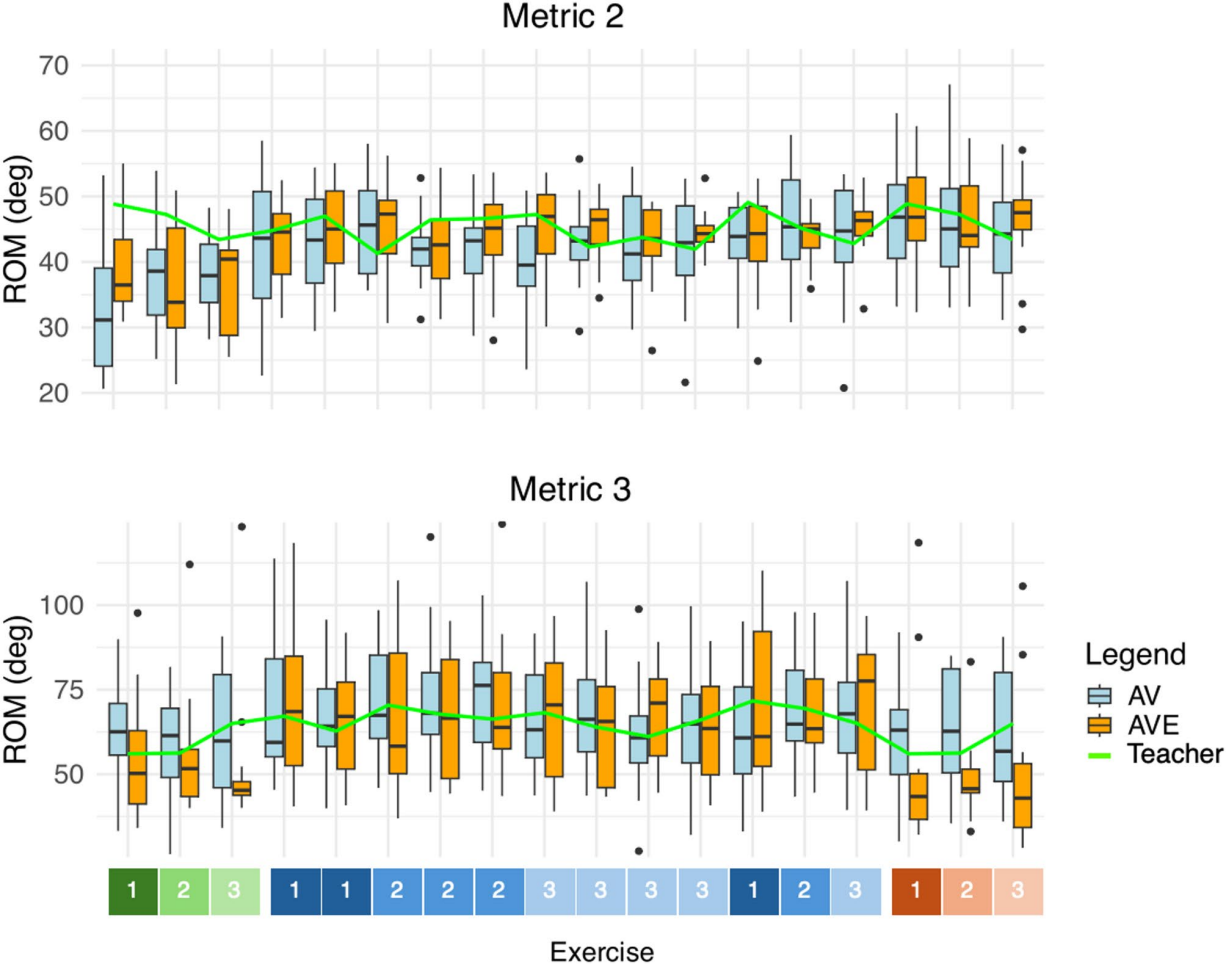
The metric values are recorded for each exercise throughout the entire experiment, including baseline, training and recall measurement. ROM elbow FE (Metric 2) and ROM shoulder AA (Metric 3) are shown. In Metric 2, performance gradually improves throughout the experiment. In Metric 3, a notable change is observed in AVE during the training measurement. The green line is the performance of the teacher throughout the experiment. Baseline (green), training (blue), recall (red). AV = control group, AVE = haptic group, deg = degrees, ROM = range of motion.

**Fig. 12:**
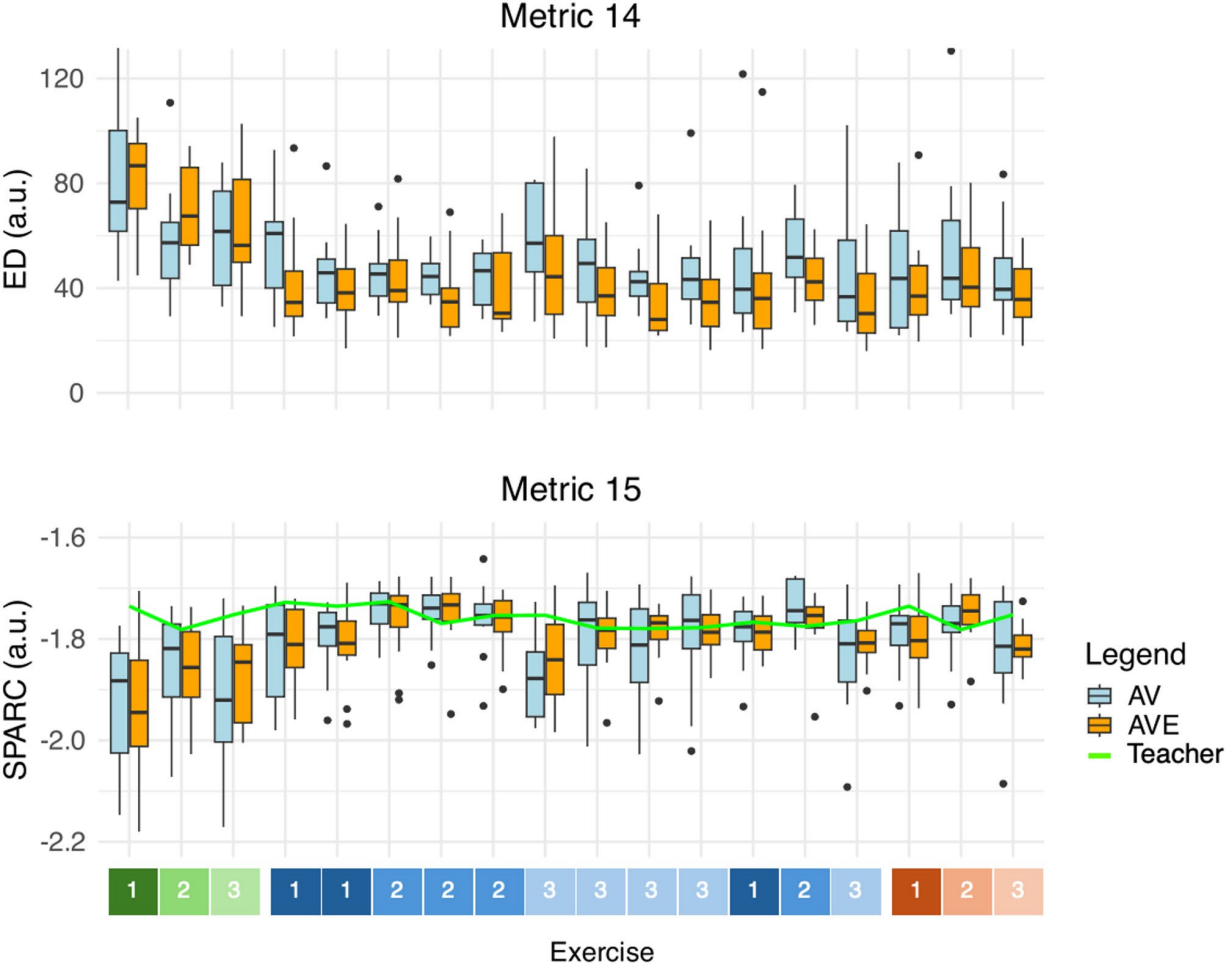
The metric values are recorded for each exercise throughout the entire experiment, including baseline, training and recall measurement. Euclidian distance (ED, Metric 14) and spectral arc length (SPARC, Metric 16) are shown. Metrics 14 and 15 gradually improve throughout the experiment. In Metric 14, a notable and sudden improvement is observed in AVE during training. The green line is the performance of the teacher throughout the experiment. Baseline (green), training (blue), recall (red). a.u. = arbitrary units, AV = control group, AVE = haptic group, deg = degrees, ED = Euclidian distance, SPARC = spectral arc length.

An overview of the recorded metrics is provided in Table 4. Contrasts of the statistical analysis are provided in Table 5. A graphical representation of the results is shown in Figs. 10, 11 and 12.

**Table 4:** Mean and standard deviations of 13 Metrics as recorded.

Metric	Units	AV		AVE		Teacher
		S1 (mean ± SD)	S3 (mean ± SD)	S1 (mean ± SD)	S3 (mean ± SD)	(mean ± SD)
2	deg	36.7 ± 8.6	45.8 ± 8.6	36 ± 11	46.9 ± 7.8	45.4 ± 2.5
3	deg	62 ± 16	61 ± 18	55 ± 20	52 ± 25	64.1 ± 5.0
4	a.u.	1.29 ± 0.59	2.7 ± 2.1	1.42 ± 0.81	2.7 ± 1.8	2.11 ± 0.37
6	a.u.	13.4 ± 3.5	22 ± 13	14.8 ± 5.9	23 ± 12	16.5 ± 2.5
8	a.u.	12.05 ± 4.37	9.7 ± 2.6	12.8 ± 7.9	8.9 ± 1.3	7.86 ± 0.26
10	deg	2.2 ± 1.1	2.3 ± 1.2	2.1 ± 1.1	1.86 ± 0.65	1.25 ± 0.23
11	deg	3.6 ± 1.9	3.7 ± 1.8	2.6 ± 1.0	2.3 ± 1.1	1.49 ± 0.38
12	deg	34 ± 34	29 ± 24	20 ± 18	17 ± 16	6.2 ± 1.8
13	s	19 ± 20	0 ± 27	6 ± 71	3 ± 14	NA
14	a.u.	66 ± 24	49 ± 23	78 ± 38	42 ± 17	NA
15	a.u.	-1.90 ± 0.13	-1.795 ± 0.086	-1.89 ± 0.12	-1.791 ± 0.065	-1.758 ± 0.019
16	a.u.	0.896 ± 0.062	0.923 ± 0.075	0.82 ± 0.20	0.935 ± 0.048	NA
17	deg	-0.07 ± 0.15	0.04 ± 0.24	-0.16 ± 0.77	-0.04 ± 0.17	NA

Spatial metrics (Metrics 2, 3, 4, 6, 8, 10, 12),Temporal metrics (Metric 13), Spatiotemporal metrics (Metrics 14, 15, 16, 17). a.u. = arbitrary units, AV =control group, AVE = haptic group, deg = degrees, NA = not applicable, S1 = baseline measurement, S2 =training, S3 = recall measurement, SD = standard deviation.

**Table 5:** Contrasts of the Group: Stage interaction for 13 Metrics from the statistical analysis. Metrics that showed a significant effect over the different stages (S1, S2, S3) are indicated in bold. Contrasts are given in the column estimate, with upper (CI.Upper) and lower (CI.Lower) credibility intervals (95%), and a posterior probability(post.prob). (AV S1 - AV S2) - (AVE S1 - AVE S2) indicates contrasts between baseline measurement and training measurement, (AV S2 - AV S3) - (AVE S2 - AVE S3) indicates contrasts between training and recall measurement, (AV S1 - AV S3) - (AVE S1 - AVE S3) indicates contrasts between baseline and recall measurement. Spatial metrics (Metrics 2, 3, 4, 6, 8, 10, 12), Temporal metrics (Metric 13), Spatiotemporal metrics(Metrics 14, 15, 16, 17). a.u. = arbitrary units, AV = control group, AVE = haptic group, CI = credibility interval, deg = degrees, S1 = baseline measurement, S2 = training measurement, S3 = recall measurement.

Metric	Units	contrast	Estimate	CI.Lower	CI.Upper	Probability
**2**	**deg**	**(AV S1 - AV S2) - (AVE S1 - AVE S2)**	**6.6**	**-1.3**	**14.7**	**0.95**
		**(AV S2 - AV S3) - (AVE S2 - AVE S3)**	**0.4**	**-7.5**	**8.4**	**0.54**
		**(AV S1 - AV S3) - (AVE S1 - AVE S3)**	**7.07**	**0.58**	**13.81**	**0.98**
**3**	**deg**	**(AV S1 - AV S2) - (AVE S1 - AVE S2)**	**3.5**	**-6.0**	**13.0**	**0.76**
		**(AV S2 - AV S3) - (AVE S2 - AVE S3)**	**-8**	**-19**	**1**	**0.05**
		**(AV S1 - AV S3) - (AVE S1 - AVE S3)**	**-4.9**	**-9.8**	**-0.5**	**0.01**
4	a.u.	(AV S1 - AV S2) - (AVE S1 - AVE S2)	0.01	-0.49	0.50	0.52
		(AV S2 - AV S3) - (AVE S2 - AVE S3)	0.23	-0.51	0.98	0.73
		(AV S1 - AV S3 - (AVE S1 - AVE S3)	0.23	-0.30	0.81	0.82
6	a.u.	(AV S1 - AV S2) - (AVE S1 - AVE S2)	-1.6	-5.2	2.0	0.18
		(AV S2 - AV S3) - (AVE S2 - AVE S3)	1.8	-3.3	6.9	0.76
		(AV S1 - AV S3) - (AVE S1 - AVE S3)	0.2	-3.6	4.0	0.54
8	a.u.	(AV S1 - AV S2) - (AVE S1 - AVE S2)	-0.3	-1.8	1.1	0.33
		(AV S2 - AV S3) - (AVE S2 - AVE S3)	0.23	-0.89	1.41	0.66
		(AV S1 - AV S3) - (AVE S1 - AVE S3)	-0.1	-1.1	0.9	0.43
10	deg	(AV S1 - AV S2) - (AVE S1 - AVE S2)	-0.19	-0.60	0.23	0.19
		(AV S2 - AV S3) - (AVE S2 - AVE S3)	-0.01	-0.45	0.43	0.48
		(AV S1 - AV S3) - (AVE S1 - AVE S3)	-0.20	-0.50	0.09	0.09
11	deg	(AV S1 - AV S2) - (AVE S1 - AVE S2)	0.01	-0.74	0.74	0.50
		(AV S2 - AV S3) - (AVE S2 - AVE S3)	-0.2	-1.0	0.5	0.27
		(AV S1 - AV S3) - (AVE S1 - AVE S3)	-0.24	-0.64	0.16	0.12
12	deg	(AV S1 - AV S2) - (AVE S1 - AVE S2)	0.1	-8.2	8.3	0.51
		(AV S2 - AV S3) - (AVE S2 - AVE S3)	0.6	-7.6	8.8	0.56
		(AV S1 - AV S3) - (AVE S1 - AVE S3)	0.7	-4.5	5.6	0.60
13	s	(AV S1 - AV S2) - (AVE S1 - AVE S2)	3.9	-6.6	14.3	0.77
		(AV S2 - AV S3) - (AVE S2 - AVE S3)	-4	-14	6	0.22
		(AV S1 - AV S3) - (AVE S1 - AVE S3)	-0.4	-7.8	7.4	0.47
**14**	**a.u.**	**(AV S1 - AV S2) - (AVE S1 - AVE S2)**	**-9**	**-20**	**1**	**0.05**
		**(AV S2 - AV S3) - (AVE S2 - AVE S3)**	**1.6**	**-8.2**	**11.4**	**0.62**
		**(AV S1 - AV S3 - (AVE S1 - AVE S3)**	**-8**	**-12**	**-3**	**0.00**
15	a.u.	(AV S1 - AV S2) - (AVE S1 - AVE S2)	-0.008	-0.086	0.068	0.42
		(AV S2 - AV S3) - (AVE S2 - AVE S3)	0.008	-0.077	0.095	0.57
		(AV S1 - AV S3) - (AVE S1 - AVE S3)	0.000	-0.055	0.056	0.50
**16**	**a.u.**	**(AV S1 - AV S2) - (AVE S1 - AVE S2)**	**0.020**	**-0.000**	**0.043**	**0.97**
		**(AV S2 - AV S3) - (AVE S2 - AVE S3)**	**-0.006**	**-0.018**	**0.006**	**0.17**
		**(AV S1 - AV S3) - (AVE S1 - AVE S3)**	**0.014**	**-0.002**	**0.033**	**0.95**
17	deg	(AV S1 - AV S2) - (AVE S1 - AVE S2)	0.012	-0.079	0.105	0.60
		(AV S2 - AV S3) - (AVE S2 - AVE S3)	-0.02	-0.11	0.08	0.37
		(AV S1 - AV S3) - (AVE S1 - AVE S3)	-0.004	-0.064	0.053	0.44

### Questionnaires

#### Self-Evaluations

**Fig. 13:**
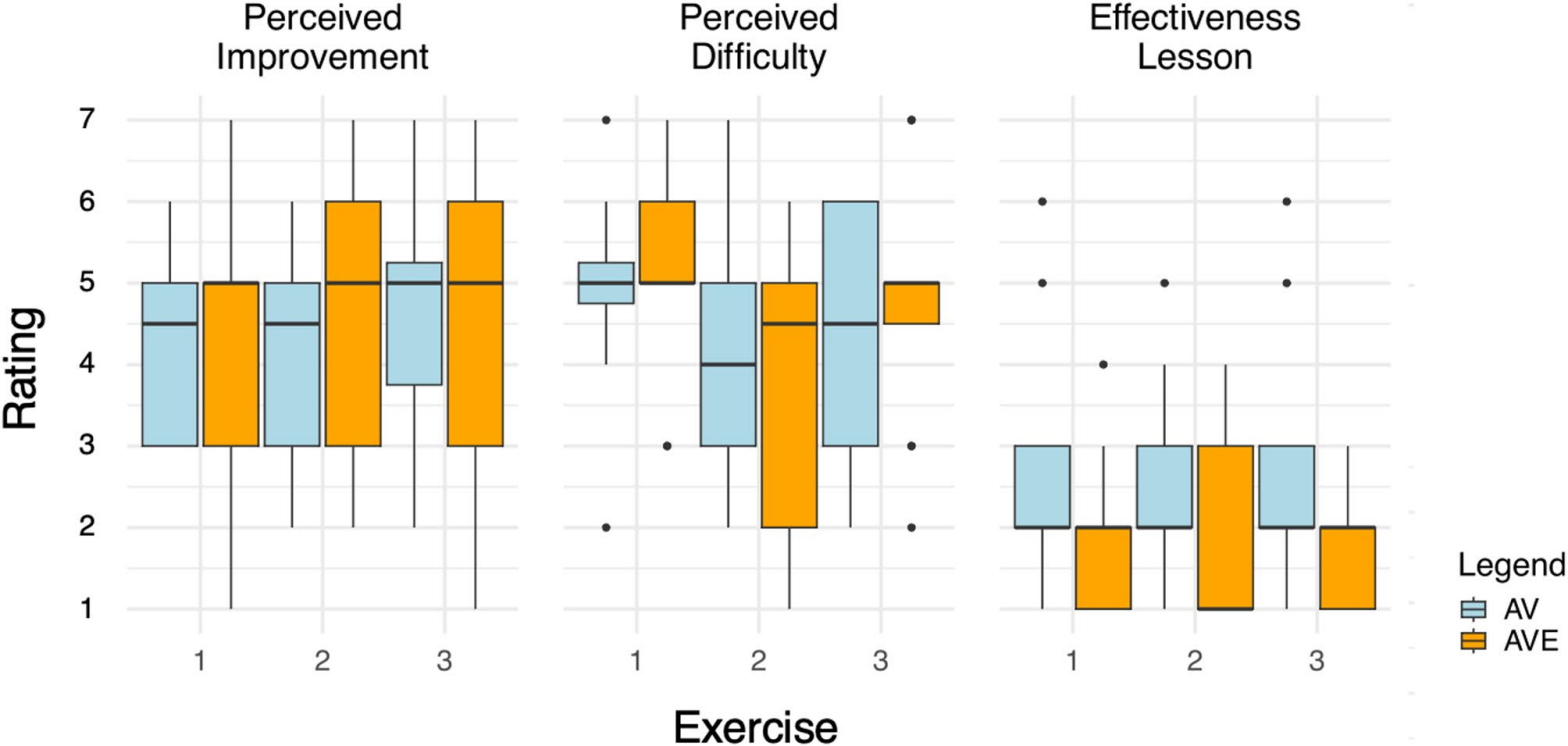
Self-evaluation ratings of the participants’ panel, with available ratings between 1 and 7. Perceived improvement: 1 = not at all, 7 = a lot); Effectiveness Training: 1 = a lot, 7 = not at all; Perceived Difficulty: 1 = very difficult, 7 = very easy.

The results of the comparisons between AV and AVE indicate that there is no difference in the self-evaluation, based on the question: “How well do you think you improved pre- versus post-intervention, in exercise x?” (a Likert rating, 1 = not at all, 7 = a lot). However, based on the question: “How well did the training instruct you how to improve exercise x?” (1 = a lot, 7 = not at all), AVE rated significantly higher, with an average difference in rating of 1.77 (HPD: [0.415, 4.08]). The groups did not rate the difficulty of the exercises differently, based on the question: “How difficult did you find exercise x?” (1 = very difficult, 7 = very easy) (Fig. 13; Table 6).

**Table 6:** Contrasts of self-evaluation assessment of the violin performances.

Perceived Improvement			
Contrast	estimate	HPD.Lower	HPD.Upper
AV - AVE	-0.40	-1.81	1.09
Effectiveness Training			
contrast	estimate	HPD.Lower	HPD.Upper
AV - AVE	1.77	0.42	4.08
Perceived Difficulty			
contrast	estimate	HPD.Lower	HPD.Upper
AV - AVE	-0.3	-1.5	0.9

Available ratings of the participants’ panel were set between 1 and 7. perceived improvement: 1 = not at all, 7 = a lot); effectiveness training: 1 = a lot, 7 = not at all; perceived difficulty: 1 = very difficult, 7 = very easy. AV = control group, AVE = haptic group, HPD = highest posterior density.

#### Prior knowledge

Participants were required to respond to 6 questions concerning their prior knowledge of string instrument playing. The inclusion criteria, as specified in the recruiting materials, requested minimal, preferably no prior knowledge about string playing. However, the questionnaires revealed that almost half of the participants possessed some prior knowledge. Based on the questionnaire results, we decided to assign participants a score to quantify their prior knowledge. Each positive response to one of the 6 questions regarding prior string instrument knowledge increased the score by 1 point. According to this criterion, the median score for AV was 1 (median = 1, IQR = 3), and the median score for AVE was 1 (median = 1, IQR = 4). Consequently, we concluded that prior knowledge was generally low and not significantly different between groups.

#### Training evaluation

Both AV and AVE found the training beneficial for learning fundamental violin movements, though with slight differences in emphasis. AV appreciated clear explanations and the opportunity to practice basic techniques but desired more specific feedback and technical explanations. Conversely, AVE found the training instructive and easy to follow, highlighting specific aspects such as arm movement and correct posture. They suggested improvements regarding more detailed instructions on holding the bow correctly and a slower pace for teaching basic concepts. Overall, both groups felt they gained familiarity with essential techniques for effective violin playing.

Both AV and AVE offered valuable insights for improving the training structure. AV suggested enhancements such as incorporating more verbal cues and instructions for holding the instrument and bow, reducing reliance on visual mirroring, and focusing more on bowing techniques. They emphasized the importance of playtime for exploration, live feedback, and post-exercise feedback, along with the need for closer attention to holding the strings and increased repetition for better learning. Conversely, AVE emphasized the importance of interactivity to check proper posture and receive feedback on bow-violin angle, as well as clearer indications of upcoming exercises for beginners. They recommended mixing different basic movements and starting with rougher movements to better understand correct arm and shoulder movements. Suggestions also included incorporating pauses, longer introductions about holding the violin and bow, and providing verbal feedback. Overall, both groups recognized the training’s strengths while offering constructive feedback for improvement.

#### Exoskeleton evaluation (Open Questions)

Participants reported various impediments while using the exoskeleton during violin practice. These included experiencing mechanical noise during certain movements, such as lifting their arms sideways, since no direct DoF was incorporated to achieve this movement. Consequently, participants were required to reach this position through a composite movement. Some found limitations in trunk movement and mobility due to the device’s design, and the requirement to stand still was noted as uncomfortable, particularly for those with existing lower back pain. Additionally, concerns were raised about the potential for damaging the device and feeling restricted in arm movement, along with feedback regarding the need for explicit and supportive assistance from the exoskeleton. These comments should be interpreted considering that the device is a prototype; thus, the overall configuration will be adjusted based on user feedback in further experiments.

Despite these considerations, participants expressed positive opinions about the exoskeleton, highlighting aspects such as the ease of feedback interpretation, assistance with movement rhythm, and correction of posture during violin playing. They appreciated the novelty and engagement with the technology, noting its potential to enhance learning experiences and provide additional guidance. However, concerns were raised about the time-consuming setup process, discomfort from wearing the device, and the perceived weight and bulkiness of the exoskeleton, which impacted overall freedom of movement and enjoyment. The short familiarization phase and the overall novelty of the device have contributed to these concerns; a longer session might improve users’ embodiment of the device and reduce discomfort as they become more accustomed to the technology.

Regarding the feedback provided by the exoskeleton, participants generally found it beneficial for executing tasks, particularly during larger movements and in guiding proper posture and timing. However, some participants felt that the feedback could be more direct, and that the device lacked subtlety in teaching specific movements. This be due to trade-offs made during the design phase, particularly the necessity of addressing the main DoFs involved in the targeted movement (bowing), specifically elbow FE and shoulder AA, which inherently limits the exoskeleton’s intervention to these joints. Despite varying perceptions of the extent of assistance provided, participants agreed that the exoskeleton added another informative dimension to learning, offering tangible support for understanding and executing violin techniques. For full survey results: see Supplementary Materials.

#### Exoskeleton evaluation (Likert Scales, 1–5)

Overall, participants reported positive experiences, finding the exoskeleton easy to operate and beneficial for enhancing their expertise and motivation in instrumental training. They generally disagreed with statements indicating difficulty or rigidity in interaction with the exoskeleton. While concerns about data tracking were not strongly expressed, participants indicated a strong intention to use the exoskeleton in future learning endeavors. For full survey results: see Supplementary Materials.

## Discussion

### Summary and key findings

In this study, 24 participants trained for 20 min by following a video lecture from a professional teacher. The AVE group received real-time haptic feedback on their movements based on those of the teacher, providing an additional information channel that could enrich their learning experience, while the AV group received no haptic guidance. We hypothesized that haptic-assisted training would result in measurable differences in learning outcomes, as assessed through participant self-assessments, evaluations by a double-blind expert panel, and a range of quantitative kinematic parameters. Using the kinematic parameters, participants’ performance was evaluated before, during, and after the training. In contrast, questionnaires were completed by participants only after the experiment, while experts rated videos of the performances recorded before and after the training measurements. By combining all these metrics, we obtain a more comprehensive understanding of the educational impacts of this technology.

Hypothesis 1 was confirmed as a whole, as expert ratings showed a moderate but significant correlation with both spatial (Metric 2, 4, 6, 8) and spatiotemporal metrics (Metric 14, 15, 16), despite a conservative correction for multiple comparisons (see Table 2). Interestingly, movement smoothness (Metric 16) correlated with expert ratings, a metric which is also involved in violin playing expressivity^43^.

The metrics that correlated with expert ratings were kept to further unravel the educational potential of the haptic-assisted training. In addition, metrics related to ROM (Metric 3), rhythm (Metric 13) and variability (Metric 10, 11 and 12) were included to evaluate the effect of the exoskeleton on kinematics, on temporal metrics, and on motor learning in general.

Based on these metrics, performance quality was measured during haptic-assisted training, thus when the haptic feedback is active in the AVE group. Importantly, since the exoskeleton only actuates elbow FE and shoulder AA, it follows that we cannot expect that all metrics associated with expert ratings will improve. Nevertheless, during training, a notable improvement can be seen in reproduction of the spatiotemporal bowing patterns as compared with the teacher (Metric 14 and Metric 16). This improvement is more pronounced in AVE, with a 40% improvement in AVE compared to an 18% improvement in AV in Metric 14, confirming Hypothesis 2.3. Additionally, a beneficial effect is visible in the ROM of elbow FE (Metric 2) in AVE as compared to AV, as the ROM increases 6.7 degrees more upon activation of the exoskeleton. The effect on this spatial metric confirms Hypothesis 2.2. However, we reject Hypothesis 2.1, as we did not find an improvement in any of the temporal metrics. Therefore, Hypothesis 2 was only partly confirmed (see Tables 4 and 5, and Figs. 10, 11 and 12).

The base levels of AV and AVE were not statistically different. Background in string playing was similar and negligible before the experiment, MSI scores did not differ significantly, and baseline qualitative metrics did not differ significantly for none of the used metrics. Consequently, participants can be considered to be novices at violin playing, thus they can be grouped in the cognitive stage of learning (e.g.,^17,22^). Given their low skill level and the challenge of the task, a distinct learning process characterized by significant improvement in performance over a relatively short time can be anticipated^27^, in both AV and AVE. In addition, there were no differences between groups in the perceived improvement of technique before, versus after training, nor in the perceived difficulty of the exercises, further reducing potential confounders (see Table 6; Fig. 13).

Therefore, it is remarkable, that based on baseline-recall comparisons between groups, the expert panel rated the performance of AVE significantly better after training, compared to AV, suggesting that the haptic feedback had a positive effect on recall (see Table 3), confirming Hypothesis 3.1.

The positive effects of haptic-assisted training were corroborated in part by the quantitative metrics. Metric 14 and 16, both spatiotemporal metrics correlating with expert ratings, improved significantly baseline versus recall in AVE as compared with AV, with a 62% improvement in AVE compared to an 26% improvement in AV in Metric 14, confirming Hypothesis 3.4. In addition, Metric 2, a spatial metric describing the ROM of the elbow FE, improved significantly in AVE relative to AV baseline versus recall, as the ROM increases 6.9 degrees, confirming Hypothesis 3.3. However, Hypothesis 3.2 was rejected, as we did not find a significant improvement in the temporal metrics. Therefore, Hypothesis 3 was only partly confirmed (see Tables 4 and 5, and Figs. 10, 11 and 12).

The beneficial effects of the haptic-assisted learning are also reflected in the outcome of the user survey: self-evaluation ratings showed a significant advantage in the perceived effectiveness of training for learning the different exercises in the AVE group (see Table 6; Fig. 13). These beneficial effects are further supported by participants’ descriptions of the added value of the exoskeleton interaction, including ease of feedback interpretation, assistance with movement rhythm, and correction of posture during violin playing (see Supplementary Materials).

This convergence of quantitative improvements, expert ratings, and participants’ subjective accounts reinforces the interpretation that haptic guidance was not only objectively effective but also perceived as pedagogically beneficial, suggesting potential for integrating exoskeleton technology into instrumental training contexts.

### Comparison with other robot-assisted music education studies

When comparing this study with other explorations in robot-assisted music education, most notably the MoveMe^29^ and HAGUS^16^ apparatuses, several similarities emerge. The MoveMe system, for example, adopted a comparable concept by using prerecorded haptic trajectories derived from an expert performance to guide students’ movements. That work demonstrated proof of principle, with real-time bidirectional feedback and suggestions for tracking progress. However, unlike the present study, MoveMe did not include quantitative motion metrics, preventing direct comparison of learning effects. Nevertheless, participants in both studies expressed a positive attitude toward haptic feedback, and the qualitative observations in MoveMe also suggested performance benefits. Similarly, results with the HAGUS device indicated a beneficial influence of haptic feedback on learning the spatiotemporal aspects of instrumental performance.

Considering the evaluation metrics for skill assessment, comparable strategies have been explored in other domains, such as surgical training, where kinematic smoothness and trajectory efficiency correlate with expert ratings of proficiency^68^. However, previous music-related MoCap studies, such as D’Amato et al. (2020)^33^,focused primarily on static skill classification rather than on longitudinal learning evaluation. A key methodological contribution of the current study, in contrast, is the establishment of a quantitative frame work that links expert evaluation with kinematic metrics to assess the learning benefits of haptic-assisted violin training. This integrative approach enabled the identification of movement features that underpin expert judgment and the use of these same quantitative indicators to track learning over time.

### Limitations in temporal metrics improvement

Although this work confirms the benefits of performance-enhancing feedback on the spatial and spatiotemporal aspects of a motor learning task^13,16^, as compared to the control group, we could not reproduce improved learning of the temporal aspects, which are nevertheless widely reported in the literature^11^.

Potentially, the rhythmical nature of the task might not be challenging enough, given the relatively high MSI scores of all participants (see Table 1). Indeed, the challenge point framework^27^ states that the provided challenge should match the skill of the participant, potentially explaining the lack of improvement of the temporal metrics.

Another potential explanation is linked to the inflated ROM of the shoulder. Indeed, the intervention of the haptic feedback seems to have a remarkable effect on the AA angles of the shoulder (Metric 3), as participants seem to increase shoulder use upon activation of the haptic feedback, with a significant increase of the ROM of shoulder AA of 3.4 degrees in AVE as compared to AV. This effect disappears when the feedback is turned off, showing even a significant difference between groups in shoulder AA between training and recall measurements, and between baseline and recall (see Tables 4 and 5, and Figs. 10 and 11). This effect can be explained due to movement restriction when the exoskeleton suit is in TM, followed by overcompensation and lack of knowledge about the correct movement characteristics when the exoskeleton suit is in HM.

Interestingly, this effect has been reported in other studies: for instance, in another study on haptic-assisted training, on a rhythmic lever manipulation task to propel a wheelchair, participants in the haptic guidance group consistently executed the arm movements with larger amplitudes than the unguided group^69^.

Consequently, it is possible, that the exoskeleton interferes with the timing of the participants, an effect also observed by Zondervan et al. (2014), who report aberrant frequencies in a timing task due to inflated movements of the haptic-guided group^69^.

A final possibility is that participants might be focused on correct execution of the bowing movements, in terms of correct use of elbow FE and shoulder AA, distracting them somewhat from the temporal aspects of the bowing movement.

Finally, the detrimental effects of haptic methods on temporal aspects of movement are also reported in work featuring performance-degrading haptic feedback^70^ or continuous rhythmical tasks^71^. However, the underlying causes are poorly understood.

### Considerations for shoulder kinematics and physical hindrances

Considering the spatial metrics, the observation that the exoskeleton induces an unnatural and potentially unwanted alteration in shoulder kinematics deserves extra attention^72^. From the rating correlations, it was inferred that shoulder kinematics only minimally correlate with expert ratings, which might explain why AVE scores higher in the qualitative ratings despite potential physical hindrances. However, the possibility that unnatural behavior is induced with potentially harmful effects in the long run must be ruled out in further iterations of this study and considered in further improvement of the device.

Potential mechanical interferences also emerged from the user feedback: participants appreciated the added value of the exoskeleton, but they also noted drawbacks such as mechanical noise, bulkiness, and occasional restrictions in movement. Integrating these experiential insights into future design iterations will be essential for translating proof-of-principle success into sustainable learning tools.

### Limitations

It is worth noting that there are some limitations in this study. Firstly, it is not clear how much of the improvement is attributable to the arousal effect (the “wow-effect”) of the technology, as reported in applications of advanced technology in education^73^. It is reasonable to assume that the acclimatization time was not sufficient to temper participants’ enthusiasm about the device. Consequently, it should be noted that this can be a confounding factor in the study.

Additionally, participants’ uncontrolled behavior during training might play a significant role in the results of motor learning. For example, like in^23^, the slight benefit of AVE over AV in learning the maximum and minimum speed of the spatiotemporal bowing trajectory might result in unforeseen behavior of participants in AV. For instance, participants might have sped up at certain points of the bowing trajectory when they observed they lagged behind the desired position, and this velocity correction might have resulted in higher speed peaks compared to those in the desired velocity profile.

Also, we presented the same video lesson to both groups, but the teacher was wearing the exoskeleton. According to the model-observer similarity (MOS) hypothesis, similarity between learner and teacher can influence learning outcomes^74^. Consequently, this confounding effect cannot be ruled out.

Additionally, the nominal task to be learned should not be considered the task per se, but rather the execution of the task with a robotic apparatus, as the use of the device alter the nominal task’s kinematics and dynamics, owing to inertia and friction effects^72^. Consequently, the haptic rendering alter the learners’ perception of the task’s goals, leading to the adoption of undesired strategies during training, as exemplified by the effect on shoulder AA. Related to these issues, the improvement after training might also be confounded by mechanisms related to motor adaptation, rather than motor learning. Further studies should be conducted to determine whether the technology’s impact was indeed detrimental to learning or positive, by enlarging the control group and anticipating prolonged use of the technology over more than a single session.

Moreover, we did not include a retention measurement at a later stage. Due to the organizational demands of this type of research, many studies in this field only assessed short-term learning with recall (catch) measurements, which were interspersed among training sessions^14,16,23^, or with immediate recall measurements right after the training^19,75,76^. However, there is evidence that short-term (within a few minutes) and long-term (24 h or more) retention can vary greatly, particularly when forms of augmented feedback have been used^77–79^. Therefore, a delayed retention measurement administered at least 24 h after training should be employed to accurately evaluate motor learning. Therefore, we can only describe the benefits of this technology over a very short timeframe.

Consequently, we did not measure to what extent participants were able to apply the learned motor skills in a new similar task, which is an important aspect of motor learning known as motor skill transfer^65^. Unfortunately, very few studies in the field of haptic-assisted training administered long-term transfer measurements on an altered version of the trained tasks^62,69^, or a real-life task^80^.

Moreover, the aim of this study was to obtain a detailed insight into the outcome of interaction with the haptic technology in a learning context, purely on the kinematic level. Therefore, we decided to show videos without sound to the experts. However, in future work, evaluation of the audio will be added to the analysis since, in this study, only kinematic aspects are evaluated.

Finally, it must be noted that the population size is limited, which constrains the generalizability of the results and is further compounded by the inclusion of only male participants. Thus, although significant effects were observed, the small sample size limits the statistical power to detect more subtle effects, particularly in temporal metrics. Future work with larger, mixed-sex cohorts and formal power analyses will be essential to confirm and extend these findings.

## Conclusion

This study is the first to explore the use of an upper-limb wearable robot for teaching violin technique to novices through haptic-assisted training. The educational potential of this technology was evaluated using a comprehensive array of quantitative and qualitative metrics, providing a detailed understanding of its learning effects and areas for improvement.

The beneficial effects of haptic-assisted learning were reflected in the assessment by a double-blind expert panel, which indicated that participants who trained with the exoskeleton outperformed the control group during a recall measurement. In addition, a selection of spatiotemporal and spatial metrics related to bowing technique improved significantly during haptic-assisted training, with persisting gains during recall. These results suggest potential learning benefits from the technology, which were also corroborated by qualitative user assessments.

The multimodal evaluation of the technology also revealed some subtle effects of haptic-assisted training. Notably, the exoskeleton appeared to interfere with natural movement patterns, particularly in shoulder ROM, warranting further investigation. Importantly, the quantitative framework linking expert judgment with kinematic metrics enabled the identification of specific kinematic features that underpin musical proficiency and allowed these indicators to track learning over time. This approach provides a transferable tool for assessing motor skill acquisition in haptic-assisted learning.

However, several limitations, including the small sample size and the lack of long-term retention measurements, caution against drawing definitive conclusions about causality.

Despite these limitations, the study provides valuable insights into the potential of haptic-assisted learning for motor skill acquisition in music education. Future research should address the identified mobility constraints, expand the sample size, and include long-term retention and transfer measurements to better evaluate the technology’s sustained impact. These next steps will be essential for refining haptic-assisted learning technology and realizing their potential to enhance motor skill acquisition across diverse fields.

## Supplementary Information

Below is the link to the electronic supplementary material.

Supplementary Material 1

Supplementary Material 2

Supplementary Material 3

Supplementary Material 4

Supplementary Material 5

Supplementary Material 6

Supplementary Material 7

## Data Availability

The datasets used and/or analyzed during the current study are available from the corresponding author on reasonable request.
