# Supplementary material for: Haptic feedback in violin education as a case study of robotic exoskeleton-mediated motor learning
Source: Sci Rep. 2026 Mar 4;16:13639. doi: 10.1038/s41598-026-39226-8 (PMC13125633; doi:10.1038/s41598-026-39226-8)
Supplement: Supplementary file 7 — Supplementary Material 7 [file 41598_2026_39226_MOESM7_ESM.pdf]

Legend Supplementary Materials:

- Ex1.mov: The teacher demonstrates Exercise 1. This video is used for baseline and recall measurements.
- Ex2.mov: The teacher demonstrates Exercise 2. This video is used for baseline and recall measurements.
- Ex3.mov: The teacher demonstrates Exercise 3. This video is used for baseline and recall measurements.
- Training.mov: The full lesson as presented to participants. The teacher demonstrates basic violin technique and the correct performance of Exercises 1, 2, and 3. This video is used for training measurements.
- Questionnaires_AVE_Group.xlsx: Contains all questionnaires used for the AVE group, including the responses of all AVE participants.
- Questionnaires_AV_Group.xlsx: Contains all questionnaires used for the AV group, including the responses of all AV participants.
